# A Systematic Review on Patient and Public Involvement in Research on Childhood Communication Difficulties

**DOI:** 10.1111/1460-6984.70281

**Published:** 2026-06-27

**Authors:** Zhixing Yang, Quynh Brooke Ho, Ria Bernard, Liam Barrett, Peter Howell

**Affiliations:** ^1^ Department of Experimental Psychology, Division of Psychology and Language Sciences University College London London UK; ^2^ Action for Stammering Children London UK; ^3^ Ear Institute University College London London UK; ^4^ National Institute for Health Research University College London Hospitals Biomedical Research Centre London UK

**Keywords:** development, developmental language difficulties, hearing difficulties, patient and public involvement, stuttering, systematic review

## Abstract

**Background:**

The proposal to involve patients and the public (Patient and Public Involvement; PPI) in research is increasingly recognised as important in research concerning speech, language, and hearing and communication difficulties. Whilst there is extensive guidance about PPI involvement by national agencies such as the National Institute of Health Research in the UK, little information is currently available concerning whether these expectations are met in any area of healthcare provision.

**Aims:**

This systematic review aimed to identify, describe and evaluate how PPI has been implemented and reported in research concerning childhood speech, language, hearing and communication difficulties.

**Methods:**

Six electronic databases were systematically searched for eligible studies reporting PPI in these fields. Records were screened against predefined eligibility criteria. Data were extracted on study characteristics, PPI contributors, stages and methods of involvement, reported impacts, and quality of PPI reporting. The methodological and PPI quality were assessed using MMAT and GRIPP2 Short Form, and findings were synthesised narratively.

**Results:**

Twenty‐one studies were included. PPI was reported variously across speech, language, hearing and communication topics, the implementations varied considerably. Parents, carers and professionals were more commonly involved than children and young people themselves. Most studies used consultation‐based approaches, often during early stages (e.g., study design or material development), while fewer reported involvement in later stages (e.g., dissemination, implementation or evaluation). Reporting was inconsistent, particularly regarding contributor characteristics, accessibility adaptations, decision‐making influence, and the impact of PPI.

**Conclusions:**

The systematic review showed that PPI in childhood speech, language, and hearing difficulties has advanced significantly, with the growing interest and number of publications, particularly in the past five years. The review also highlighted challenges such as confusion and heterogeneity of PPI concepts, representativeness issues, tokenistic practices, lack of support given to lay contributors, and limited impact evaluation. Future research should distinguish PPI from research participation, involve children and young people more directly where appropriate, report contributors’ roles and influence more transparently, and consider accessibility and communication support throughout the involvement process.

**WHAT THIS PAPER ADDS:**

*What is already known on the subject*

- Health service providers and research funders now require attention to Patients and Public Involvement (PPI) in healthcare research. PPI highlights the need to work jointly with stakeholders at all steps involved in research to allow them to take governance over their health conditions. Whilst emphasis on PPI applies to health issues in general, there is no specific review of PPI considerations in research on health factors that affect hearing and communication difficulties. This omission is particularly noteworthy when considering issues that arise in early development.

*What this study adds to existing knowledge*

- A systematic review was conducted to assess current progress with respect to PPI for hearing and communication research in childhood and adolescence. The review identified research involving PPI that met quality requirements. Challenges related to PPI were highlighted in further analyses that revealed confusion and heterogeneity in PPI concepts, representativeness issues, tokenistic practices, a lack of support given to lay contributors, and a limited evaluation of the impacts of PPI.

*What are the clinical implications of this work?*

- PPI involvement in clinical research for communication and hearing difficulties should have a positive impact on the well‐being of children and adolescents. Four challenges were highlighted when considering the current state of research on PPI in this domain: shallow involvement of the public and patients, conflicting expectations between groups participating in research, lack of clarity of research outcomes as they relate to PPI and variable standards of reporting PPI findings across studies. Tentative recommendations concerning how each of these matters could be addressed are offered.

## 1 Introduction

### 1.1 The Emergence of Patient and Public Involvement

The active involvement of patients and members of the public in the research process has been promoted internationally in response to concerns about the quality of service delivery and patient safety (Francis 2013). These concerns have grown in importance as part of a broader movement towards person‐centred care. In the UK, Patients and Public Involvement (PPI) is a broad umbrella term used to refer to this area and highlights the need for a symbiosis between healthcare and social research as well as stakeholders’ own governance over their health conditions (Boote et al. 2015). According to INVOLVE, the English national advisory group that supports active public involvement, PPI is conceptualized as ‘*research being carried out ‘with’ or ‘by’ members of the public rather than ‘to’, ‘about’, or ‘for’ them*’ (NIHR 2021a). In this context, patients and public members include any individuals who have accessed healthcare services, their carer's, and any organisational or community representatives whose lives may be impacted by the research and service delivery (NIHR 2021a).

Similar initiatives to PPI occur across the globe. It is worth noting at the outset that different terminologies are often used interchangeably to refer to similar concepts across countries and in different research contexts (Biddle et al. 2021; Hassett et al. 2024). While terms such as PPI are commonly used in the UK, similar approaches may be referred to as patient engagement in the US, or consumer and community involvement in Canada (see Hoddinott et al. 2018). Despite these terminological differences, the underlying principle remains the same, which is to ensure that research is person‐centred and addresses real‐world needs possessed by the target population *and* the public in clinical practice. By doing this, the relevance, quality, and impact of the research is enhanced (Molloy et al. 2019).

The importance of PPI has also been strongly endorsed by many governmental research funding bodies. In the UK, the National Institute for Health Research (NIHR) has played a crucial role in providing theoretical frameworks and strategic support to help researchers integrate PPI into publicly‐funded research (NIHR 2024a). Strong policy support for PPI is also seen internationally, particularly in high‐income Western countries. For example, Canada's Strategy for Patient‐Oriented Research (SPOR) and Australia's National Health and Medical Research Council (NHMRC) have all promoted co‐production of research with patients. In the US, the Patient‐Centered Outcomes Research Institute (PCORI) not only funds but also mandates patient engagement in research. Across Europe, PPI is supported through the Horizon Europe programme and various national agencies. This selection reflects this growing international policy momentum for research involving PPI components. This movement has expanded substantially in recent decades as shown in a systematic review that reported that two‐thirds of the studies featuring collaborative research components were published after 2020 (Hassett et al. 2024).

While PPI has been dramatically expanded and funded, this significant growth in PPI‐related research should be approached with caution when considering its impact. For instance, previous reviews have highlighted inconsistencies in how PPI and related frameworks are conceptualised and applied across studies, including NIHR or INVOLVE guidance on public involvement (NIHR 2024b), the GRIPP2 checklist (Staniszewska et al. 2011; Staniszewska et al. 2017), co‐production frameworks, participatory action research, and broader stakeholder or patient engagement approaches, often leading to varying levels of implementation (Biddle et al. 2021; Dawes et al. 2024; Hassett et al. 2024; Hammoud et al. 2024). It is therefore essential to assess the evidence where PPI is involved in study designs and to ascertain the extent to which research studies meet requirements inherent to PPI initiatives.

### 1.2 Is it Worth it? Evidence on PPI

Research involving stakeholders with lived experience is framed according to two broad justifications: the ethical and the consequentialist perspectives (Brett et al. 2012; Crocker et al. 2018). From a moral and ethical stance, patients and members of the public whose lives are directly impacted by research outcomes have the right to influence what research is conducted and how publicly funded research should be carried out (Brett et al. 2012; Clamers 1995; Entwistle et al. 1998). This is particularly relevant within publicly‐funded healthcare systems such as the UK's National Health Service (NHS). For service users, PPI provides an opportunity to contribute valuable insights and generate user‐relevant research based on their lived or living experiences. For researchers, incorporating patients’ input helps to ensure that the research is conducted ethically and appropriately, thereby promoting the safety and well‐being of the target patient population (Suri et al. 2024). Epistemologically, PPI enables stakeholders with lived experience to contribute unique, context‐specific knowledge, particularly concerning unmeasurable or intangible aspects of the research topic that are either overlooked or unexplored. These insights can complement academic expertise, enhancing the relevance, validity, and clinical applicability of research findings (Liabo et al. 2022).

From the consequentialist perspective, research quality, impact, and effectiveness can theoretically be enhanced via genuine partnerships between patients and researchers. Several systematic reviews that have examined studies with PPI components have highlighted the positive effects of involving patients and the wider public throughout the research process, from identifying research priorities to disseminating findings (Brett et al. 2010; Brett et al. 2014; Brett et al. 2016; Staley 2009). Additionally, the benefit of PPI on patients has also been identified and reported in these reviews, where those patients participating reported a sense of empowerment. Furthermore, quantitative evidence has also confirmed the merits of PPI. For instance, a UK‐based study found that mental health research incorporating PPI was significantly more likely to achieve successful participant recruitment than research that did not include PPI considerations (Ennis & Wykes 2013). What is more, a meta‐analysis of seven randomised studies highlighted similar results, showing that PPI was associated with increased odds of recruitment, although no significant effect was found for retention, which was possibly due to the limited number of studies available (Crocker et al. 2018). Overall, the findings emphasise the potential value of involving public stakeholders throughout the research process.

### 1.3 Challenges of PPI

Despite the broad endorsement of PPI, various challenges and criticisms coexist alongside its benefits. First, the scientific rigor of PPI has been challenged. Researchers have raised concerns regarding the inconsistent quality and poor reporting of PPI, variability in implementation, and a lack of robust evaluation methods (Edelman and Barron 2016; Hammoud et al. 2024). These issues may hinder both researchers and public members from effectively assessing the value of PPI. Second, from a practical standpoint, involving service users in research may increase the time and resources needed for planning and design, building rapport, and providing training to enable meaningful involvement by lay audiences (Brett et al. 2014; Edelman and Barron 2016). Finally, given that PPI has become an essential requirement in public funding applications, it is unsurprising that concerns have been raised about its tokenistic nature, where PPI is treated as a ‘box‐ticking’ exercise rather than a genuine partnership aligned with the principle of ‘*nothing about us without us’* (Charlton 1998). Such superficial engagement risks limiting the genuine and measurable impact of PPI on research decisions (Gibson et al. 2012; Martin 2008).

To date, most studies and reviews evaluating PPI‐related research have focused on general health and biomedical contexts without age specification (Brett et al. 2010; Brett et al. 2014; Brett et al. 2016). Hence, the generalisability of findings to inform the tailored implementation strategies is limited, especially in paediatric research. Greenhalgh et al. (2019) reviewed existing frameworks for supporting PPI implementations and concluded that a ‘one‐size‐fits‐all’ approach may hinder successful and meaningful involvement in different research contexts. Indeed, research involving children and families differs significantly from adult‐focused studies in terms of ethical considerations, goals, and the nature of involvement (Bate et al. 2016; Molloy et al. 2019). Additionally, children with different conditions often face unique barriers to effective involvement and are likely to be excluded from PPI activities. It is clear that PPI practices that do not address the specific needs of targeted child patients also do not align with the key principle of PPI (NIHR 2021a). Thus NICE, the National Institute for Clinical Excellence in the UK, has published guidance that distinguishes between involving children and young people versus adults and carers, highlighting the importance of engaging children directly and prioritising their views rather than relying solely on proxy input via, for instance, parents (NICE n.d.). Generally, discipline‐specific PPI evidence for children's health is needed, which can support the development of more tailored guidelines and context‐sensitive policies, thereby enhancing the quality, acceptability, and impact of PPI within narrower fields such as communication difficulties.

### 1.4 PPI in Childhood Communication Difficulties Research

Any limitations on communication ability, such as the literacy level achieved at different ages, can hinder making effective contributions to the research process and represents a universal barrier to involvement in research (Mitchell et al. 2019). The communication barriers are more significant for children living with communication difficulties including speech, language, and hearing (SLH) difficulties than for other health conditions.

Historically, research in communication difficulties has been predominantly expert‐driven, with limited input from patients or their families. However, in the last decade, PPI has drastically increased within the field of communication difficulties (Dawes et al. 2024; Hassett et al. 2024). For instance, Dawes et al. (2024) identified five case studies that incorporated PPI components in hearing research conducted at the Manchester Hearing Institute. Their review documented the research team's efforts to provide inclusive support, such as assistive technology (e.g., hearing loops) and interpreters for British Sign Language (BSL) users. This illustrates the need to adapt PPI processes to meet the accessibility requirements of contributors. In a narrative review, Hassett et al. (2024) explored collaborative approaches in speech‐language pathology and reported significant variability in the methodologies, reporting practices, and stages of involvement across PPI initiatives. The authors advocated for greater consistency in collaborative methodologies and the inclusion of comprehensive stakeholder engagement across all phases of research. They also highlighted a key gap whereby children and families affected by communication and swallowing difficulties were infrequently included as PPI contributors. However, these reviews have mainly focused on broad health contexts or adult populations. Less is known about how PPI is implemented and reported in childhood speech, language, hearing and communication research, where communication access, developmental stage, and proxy involvement with parents or other professionals (e.g., teachers or SLTs) may create additional challenges.

Childhood communication difficulties represent a significant public health concern due to their adverse effects on daily functioning and their potential long‐term impacts extending into adolescence and adulthood (Langbecker et al. 2020). The consequences of such difficulties often range beyond the individual, affecting key communication partners including parents, teachers, and speech‐and‐language therapists (SLTs). These multidisciplinary interactions and the diversity of lived experiences represent a complex landscape in which effective PPI requires the integration and balance of insights from multiple stakeholders. In 2015, the Communication Trust, a coalition of over 50 not‐for‐profit organisations in England supporting children and young people with Speech, Language, and Communication Needs (SLCN), published guidance emphasizing the importance of involving children, young people, and their parents in decision‐making processes to ensure that the child's voice remains central. All this evidence suggests that it is an appropriate time to examine the status and quality of PPI‐related childhood communication difficulties (including SLH difficulties) by systematic review, bearing in mind that it is a decade since guidance on PPI was published (NIHR 2024).

### 1.5 The Current Study

To our knowledge, although related reviews have examined PPI and collaborative approaches in various health research (Dawes et al. 2024; Hassett et al. 2024; Hoddinott et al. 2018; Totzeck et al. 2024), no systematic review has rigorously evaluated PPI in research concerning speech, language, hearing, and communication conditions in children and adolescents. Given that communication is essential to social participation and day‐to‐day activities, children with communication difficulties may face heightened risks of adverse socioeconomic, psychological, and educational outcomes (National Academies of Sciences, Engineering, and Medicine 2016). It is therefore crucial to involve stakeholders’ ideas in the research process to better inform identification, early intervention, and prognosis.

In this review, speech, language and hearing disorders are considered health‐related conditions and therefore fall within the scope of PPI as a concept rooted in health and clinical research. However, we recognise that research concerning childhood speech, language and hearing often spans across other disciplines (e.g., community settings), and that not all lived‐experiencers may identify with medicalised terms such as ‘*disorder*’ (e.g., Constantino et al. 2022). This further highlights the importance of PPI, as contributors can help researchers reflect on whether the language used to describe communication differences, difficulties or disorders is acceptable, accurate and meaningful to those affected. Related terms such as participatory research, co‐production, stakeholder engagement and user involvement were therefore included in the search strategy to capture relevant involvement practices across this multidisciplinary field. Studies were included only where children, families or public contributors helped shape the research process or outputs, rather than solely contributing data as research participants.

The present review aimed to systematically examine the status and quality of PPI in research on childhood communication difficulties, which includes childhood SLH difficulties. A secondary aim was to assess the impact of PPI on research design, implementation, and dissemination, as well as its broader contributions. Finally, the findings were used to formulate evidence‐based recommendations to support meaningful PPI integration in future research within the field of childhood communication difficulties.

## 2 Methods

### 2.1 Protocol Registration

The current review was pre‐registered on PROSPERO before the review process started (Registration number: CRD42024521073). This systematic review was reported in accordance with the Preferred Reporting Items for Systematic Reviews and Meta‐Analyses (PRISMA) 2020 statement (Page et al. 2021). A completed PRISMA checklist is provided in Appendix A.

### 2.2 Search Strategy

Two reviewing rounds were conducted to ensure an up‐to‐date and comprehensive search: The first took place from March 2024 to August 2024, and the second was conducted in February 2025. To acquire a comprehensive search, the systematic searches were conducted on six bibliography databases: MEDLINE (Ovid version); PsycINFO (Ovid version); EMBASE (Ovid version); Cochrane Library; CINAHL and Web of Science; and Linguistic and Language Behaviour Abstracts (LLBA). Since out current review focuses on the peer‐reviewed publications to maintain methodological rigour and comparability across studies, grey literature was excluded. The search strategy was developed and finalised in cooperation with experts in PPI and professionals in childhood SLH difficulties based at UCL.

Search terms were developed for three aspects: 1) PPI, 2) SLH difficulties/disorders/impairments, and 3) childhood and adolescence. Due to the massive heterogeneity of PPI concepts used across different research contexts, in this review, to avoid inconsistency, PPI is conceptualised as lived‐experience involvement, in line with NIHR (2021b). NIHR clearly distinguishes between participation and involvement: participation refers to individuals taking part as research subjects (e.g., passively providing data without informing the research decisions), whereas involvement refers to lived‐experience patients and members of the public actively contributing to various stages of the research process, including its design, conduct, analysis, and dissemination. In the present review, PPI was conceptualised as the involvement of children and adolescents with lived, or living, experience to any types of communication difficulties, and of their key communication partners. The key communication partners have caregiving and managing experiences with the difficulties, including parents, professionals such as teachers and clinicians, members of the public, and community support networks. Parent/carer involvement was therefore distinguished from, but not treated as a replacement for, the direct involvement of children and young people. Studies involving only professionals were excluded unless professionals were involved alongside children, parents, carers or other public contributors. They should be involved in the research cycle to shape the research decisions or outputs as stipulated by NIHR (2021a).

To include potentially eligible studies at a global level, the search incorporated terminologies used to describe PPI across countries (e.g., Patient‐centred Outcomes as used in the US, and Consumer and Community Involvement in Australia (Hoddinott et al. 2018)), given the various wordings referring to the PPI concept and to capture international work.

Although it was intended to report results separately for speech and hearing difficulties, the paucity of PPI studies meeting quality criteria precluded this. Title and abstract of each article retrieved were screened for potentially relevant papers, followed by a full‐text screening based on predetermined eligibility criteria. The search was conducted according to Medical Subject Headings (MeSH), title and abstract. Reference lists of included studies and any relevant systematic reviews were manually screened to avoid missing potentially eligible papers. The search was limited to reports in English.

### 2.3 Eligibility Criteria

The eligibility criteria were developed in accordance with the PICOS framework (Center for Reviews and Dissemination 2009), which was adapted to address the aim and topic of the current review. The inclusion and exclusion criteria are summarised in Table 1. Published peer‐reviewed papers focusing on childhood speech, language and hearing difficulties that contain primary‐sourced data incorporating PPI elements in their studies were eligible for the current review, as they adhere to established academic standards for reporting and dissemination. This consistency enables a more systematic and comparable assessment of how PPI contributions are described, valued, and integrated across studies. Moreover, peer‐reviewed publications represent the primary platform through which researchers communicate methodological approaches and findings to the wider scientific community, making them particularly relevant for evaluating the reporting of PPI in research practice. The first stage of the study selection process involved screening the titles and abstracts of each paper to exclude any papers that failed to meet the inclusion criteria shown in Table 1. The second stage of the study selection process involved examining the full text of the potentially relevant papers and excluding papers that fulfilled the exclusion criteria listed in Table 1.

**TABLE 1:** Eligibility criteria for the search.

Inclusion criteria	Exclusion criteria
Population	
Children and adolescents (age 3–17) with childhood audiological impairment or hearing problems, or speech and language difficulty as the target research population.	Less than 50% of the target research population were children or adolescents aged 3–17 years.
Type of intervention	
Any studies with patient and public involvement (PPI), as defined by the NIHR (2021b). The involvement does not refer to patient and public members being research participants, but rather being involved in any stages of the research process.	Do not contain any activities aligning with the NIHR INVOLVE definition of PPI (e.g., qualitative study without involving participants taking more active and flexible roles, Mc Menamin et al. 2022).
Control	
N/A	N/A
Main outcomes	
Study containing components and criteria meeting PPI standards.	Do not report PPI components.
Condition or domain being studied	
Patient and public involvement. Childhood speech and language difficulties. Childhood audiological impairment and hearing loss.	Any other conditions or speech delays instead of the stipulated difficulties.
Language	
Full text available in English.	Not in English.
Type of study	
Any study with primary‐sourced data using quantitative, qualitative, mixed‐method, cross‐sectional, and observational designs.	Are not peer‐reviewed and published, case reports, conference abstracts, theses or dissertations, protocols, and review articles.
Context	
Any organisations providing the space for PPI meetings between academics, people with lived experience, and public members (e.g., university, primary care, hospital).	

*Note*: The age criterion refers to the target research population of the included studies, rather than the age of PPI contributors. PPI contributors could include children and young people themselves, as well as parents, carers, teachers, clinicians, community representatives or other key communication partners.

### 2.4 Study Selection

Search results from electronic databases were deduplicated using EndNote (Niles Software 2023) and then imported to Rayyan (Ouzzani et al. 2016), which was used for further screening of eligible studies. Two reviewers (ZY and BH) independently assessed all studies before discussing any discrepancies. The inter‐rater reliability in decisions for study selection was 98.6%. A consensus of study inclusion was achieved between the two reviewers by discussions. Any continuing disagreements were resolved by consulting with the corresponding author (PH).

### 2.5 Data Extraction

Data from eligible studies was extracted and recorded using a pre‐developed worksheet in Microsoft Excel. Table 2 gives the categories and descriptions of details extracted from eligible studies. Selected studies were divided equally between two researchers (ZY and BH) for independent data extraction and later merged and reassessed by both researchers.

**TABLE 2:** Extracted information from the included studies.

Category	Description
**Study‐related information**	
Author(s)	Author(s) of the study
Year of publication	
Country	Country of publication
Study design	Data collection method(s)
Research question(s)	
Participant characteristics	Characteristics of participants (e.g., sample size, age)
Type of difficulties	Type of speech and language difficulties and hearing‐related difficulties of interest
Confirmation of difficulties	Methods to confirm diagnosis of the difficulty/difficulties
Comorbidities	Dual diagnosis besides SLD or hearing‐related difficulties
**PPI‐related information**	
PPI term	Term used to refer to PPI
PPI contributors’ characteristics	Characteristics of participants (e.g., sample size, age)
No. of PPI contributors	Number of PPI contributors
Recruitment	Recruitment method of PPI contributors
Compensation	Compensation for PPI contributors
PPI activities	Specific PPI activities
Impact of PPI	Influence and results of PPI on the study, either positive or negative
Evaluation of PPI	Critically reflect on PPI process, including limitations and strengths
Clinical implication of PPI	Suggestions and impacts of PPI to the broader population, research, or clinical practice

### 2.6 Methodological Quality Assessment

The methodological quality of each study was assessed using the Mixed Methods Appraisal Tool (MMAT) (Hong et al. 2018). MMAT is a critical appraisal instrument for systematic reviews that include qualitative, quantitative, and mixed methods designs. Based on the total score, qualitative and quantitative studies would be classified as ‘low’, ‘moderate’ or ‘high’ quality if they were scored 1–2, 3, and 4–5, respectively. Fifteen criteria were used to evaluate studies with a mixed methods design, categorizing them as ‘low’ (1–7), ‘medium’ (8–10), and ‘high’ (11–15). The methodological quality assessment was used to provide context for the interpretation of findings from each study.

### 2.7 Synthesis of Findings

Due to the descriptive nature of the current research topic, a narrative synthesis was adopted to summarise and explain data extracted from included papers. The process of narrative synthesis followed ESRC (Economic and Social Research Council) guidance (Popay et al. 2006). This approach was chosen because of the robustness of the ESRC guidance, which allowed for the methodological heterogeneity across selected studies. Following preliminary synthesis of the findings from included studies, each PPI characteristic was coded inti a short phrase (e.g., just compensations). The codes that shared a similar concept were refined and grouped into meta categories (e.g., lack of support to PPI contributors) based on an iterative process. Then, the meta categories were assigned to overarching synthesis domain to describe the nature of PPI components (e.g., tokenism). Following this, the relationships in the extracted data were explored by mapping the concepts suggested in the ESRC guidance.

This was not a formal thematic analysis of primary qualitative data. Instead, extracted study‐level information was grouped into synthesis domains to describe recurring patterns in how PPI was implemented, reported and evaluated across the included studies.

## 3 Results

### 3.1 Study Characteristics

**FIGURE 1:**
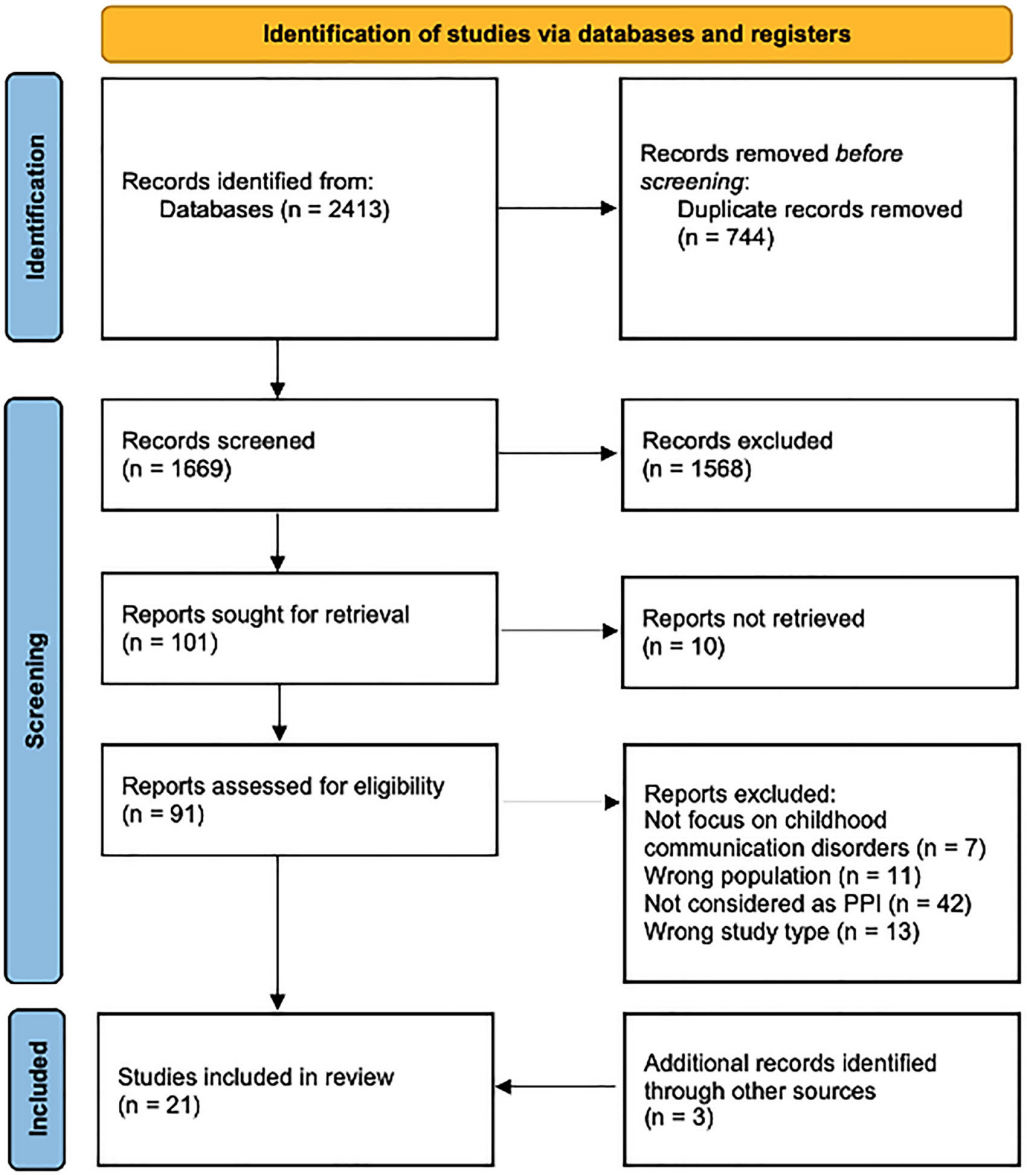
PRISMA flowchart.

The PRISMA flowchart summarising the study selection procedure is given in Figure 1. Overall, 21 peer‐reviewed publications identified from the electronic databases were included in the current review, with nine papers about speech and language difficulties, eight regarding hearing‐related difficulties, and four papers related to general communication difficulties. Among these papers, five papers used a quantitative design, seven papers used qualitative methods, and the remaining nine papers employed mixed‐method designs. Detailed characteristics of each included study are given in Table S1.

Six papers (Berquez et al. 2011; Biggs and Hacker 2021; Cooke and Millard 2018; Julien et al. 2021; Nielson et al. 2020; Singer et al. 2020) reported PPI as the basis of their study by implementing PPI as their methodology. Other studies reported PPI as a component complimenting or facilitating the research. Among the first six papers, three used the Delphi approach, which aligns with the principles of PPI as it allows stakeholders’ voices to be heard using a systemic funnel approach.

Some studies involved participants who had a co‐occurring diagnosis of anxiety disorder (Kishida et al. 2022), Down syndrome (Hall et al. 2019), cleft palate (Sweeney et al. 2020) and other developmental difficulties or disabilities (Biggs and Hacker 2021). All 21 studies were conducted between 2000 and 2024. Seven studies were published up to 2020 and the remaining 14 published from 2020 onwards. Countries of origin were the United Kingdom (*n* = 9), the United States (*n* = 4), Ireland (*n* = 2), Canada (*n* = 1), Spain (*n* = 1), the Netherlands (*n* = 2), Denmark (*n* = 1), and Australia (*n* = 1).

### 3.2 Methodological Quality Assessment

Quality was high for *n* = 15, medium for *n* = 5, and low for *n* = 1 (summarised in Table S1). Most studies clearly stated their research questions and described data collection methods and how data were gathered. Methodological components that resulted in a low or medium quality involved insufficient interpretation and analyses of data or flaws in data collection procedures (e.g., participants were not blinded about their treatments, Sweeney et al. 2020), and high non‐response rates (e.g., over 50% in Munoz‐Baell et al. 2008). Eight studies assumed SLH diagnoses based on recruitment channels without confirming the diagnoses. This may have compromised the validity and reliability of findings by increasing the risk of sampling bias, misclassification, and misinterpretation of results (Althubaiti 2016).

### 3.3 Synthesis of Findings

Findings were analysed narratively due to the descriptive nature of the current research reports. Four synthesis domains were generated to group extracted data from the included studies into the topic that highlight key patterns about the nature, implementation, and impact of PPI in research focusing on children's communication difficulties. These were: 1) Diversity and Representation in PPI Contributors; 2) PPI Activities and Roles; 3) Cycle of Tokenism; 4) Evaluations on the Impact of PPI. Details of the extracted PPI data from the 21 included studies are summarised in Table S2 (supplementary information).

### 3.4 Diversity and Representation of the PPI Contributors

#### 3.4.1 Characteristics of PPI Contributors

Consistent with the eligibility criteria, parents and carers were treated as key communication partners with relevant caregiving and proxy experience, whereas studies solely involving professionals, including researchers, speech‐language therapists or pathologists, clinicians, paediatricians, or other professionals specialising in SLH difficulties and paediatrics, were not considered lived experience involvement. When professionals collaborated with individuals with lived experience or the public contributors, their contributions were then considered as a part of a broader PPI framework where the collaborative input is enriched by both perspectives. Studies that included these met the threshold for PPI because they centre on the lived realities of affected individuals and families in the research process. The number of PPI contributors ranged from three to 154 PPI contributors across studies, where the remaining three studies (Bernard and Norbury 2023; Munoz‐Baell et al. 2008; Sweeney et al. 2020) did not report the total number of PPI contributors. It is worth noting that larger numbers generally reflected broad consultation activities, such as Delphi rounds, usually with the aims of identifying and prioritising, rather than intensive collaboration or co‐production. Therefore, contributor number was interpreted alongside the format, stage and level of involvement, rather than as an indicator of PPI depth or quality. PPI contributors varied from different lived, caregiving, or managing experiences of childhood SLH.

Overall, eight studies involved children and young people with communication difficulties during the research procedures (Alsebayel et al. 2024; Bernard and Norbury 2023; Berquez et al. 2011; Buckeridge et al. 2019; Cooke and Millard 2018; Gallagher et al. 2019; Julien et al. 2021; Kishida et al. 2022). In some papers, researchers invited people with lived experiences of other concurrent conditions that affected communication outside these age groups to participate, although the overall research focus remained on children. Examples included adults with Down Syndromes (Hall et al. 2019) or young adults with language difficulties (Singer et al. 2020), disabled students (Wilkinson et al. 2021), and college students who were involved in designing guidelines to develop a first concept the study (Singer et al. 2022).

Instead of directly involving children and adolescents with communication difficulties, most of the included studies invited parents or family members of children and young people with communication difficulties as a whole or part of PPI implementation (Berquez et al. 2011; Biggs and Hacker 2021; Francis et al. 2018; Gallagher et al. 2019; Hall et al. 2019; Julien et al. 2021; Nielson et al. 2020; Singer et al. 2020; Singer et al. 2022; Sweeney et al. 2020; Vickers et al. 2021; Wilkinson et al. 2021; Wischmann et al. 2024). In one study (Singer et al. 2020), the authors outlined their rationale for not involving children with language difficulties in the PPI activity. Singer et al. (2020) instead invited parents to contribute because: 1) the linguistic and cognitive complexity of the subject hindering children's involvement; and 2) young adults involved in the consultations expressed their feelings of discomfort when discussing a definition with researchers. Additionally, some studies also reported the involvement of public members, such as teachers and charity worker representatives.

Notably, studies with more than 13 contributors were more likely to rely on broader consultation‐based approaches, including surveys or Delphi rounds, whereas smaller groups were more often involved through iterative discussion, advisory input or co‐design activities. This suggests a trade‐off between breadth of representation and depth of involvement. Table 3 summarises the types of PPI contributors in the selected papers.

**TABLE 3:** Characteristics of PPI contributors.

Type of PPI contributors	Number of studies
Parents, carers, family members	12
Children and adolescents with SLH difficulties	8
Community and organisation representatives, members of school boards, guidance counsellors	6
Mainstream/classroom teachers	5
College students	1

#### 3.4.2 Demographic Diversity

Although some studies reported some demographic information about PPI contributors, such as whether most of the PPI contributors were female and whether they lived in urban environments (e.g., Gallagher et al. 2019), most studies failed to document this information.

#### 3.4.3 Recruitment Methods Applied With PPI Contributors

PPI contributors were primarily recruited through healthcare services, specialist clinics, and via community or public resources. Recruitment in some reports was based on the pre‐existing connections, such as parents who had already called on the service providers. Some studies that focused on children and adolescents with hearing‐related diagnoses recruited PPI contributors from designated schools, colleges, or special education programs. Four studies did not mention their recruitment methods (Buckeridge et al. 2019; Francis et al. 2018; Hall et al. 2019; Sweeney et al. 2020). Recruitment methods are summarised in Table 4.

**TABLE 4:** Sources of recruiting PPI contributors.

Type	Description	Examples
Healthcare services	Audiology	ENT & Audiology Department of Copenhagen Hearing and Balance Centre (Wischmann et al. (2024); Local and regional hearing healthcare clinics (Studts et al. 2022)
	Speech‐language‐hearing services	Michael Palin Center for Stammering Children (Berquez et al. 2011; Cooke and Millard 2018)
	Childhood disabilities	PenCRU Family Faculty (Wilkinson et al. 2021)
	Paediatrics	Primary Children's Hospital (Nielson et al. 2020)
	SLT practitioners	Singers et al. (2022)
Schools	Special education	Schools for deaf and hard‐of‐hearing children Vickers et al. (2021).
	Mainstream	Christopulos and Redmond (2023); Kishida et al. (2022); Vickers et al. (2021).
	Local colleges	Alsebayel et al. (2024); Singer et al. (2022); Wilkinson et al. (2021).
Public resources	Community resources	Social media (Biggs and Hacker 2021; Singer et al. 2020).
	Charities	Charity meeting (Vickers et al. 2021); Action for Stammering Children (Bernard and Norbury 2023).
	Support networks	British Stammering Association website (Berquez et al. 2011); Spanish Confederation of the Deaf (Munoz‐Baell et al. 2008); national support network for parents with DLD (Gallagher et al. 2019).

### 3.5 PPI Activities and Roles

#### 3.5.1 Characteristics and Format of PPI Activities

PPI contributors were involved in various PPI activities performed in a wide range of formats depending on different research aims and available resources, which included focus groups, consultations, semi‐structured interviews, or surveys for information acquisition. These methods were interpreted as PPI only where they were intended to be used to inform research decisions or outputs, rather than as data collection for the primary empirical research question. Thus, PPI contributors participated in passive data collection via surveys when researchers aimed to get wide views of large groups of stakeholders (Berquez et al. 2011; Cooke and Millard 2018; Munoz‐Baell et al. 2008; Singer et al. 2020) and for ease‐of‐collection purposes (Wischmann et al. 2024). In some studies, one‐off or periodical consultations with PPI contributors were conducted. When it was deemed necessary to acquire a deeper level of knowledge towards a specific subject, semi‐structured interviews were conducted. These provided qualitative data, which were analysed and used to advocate aspects of the decision‐making process. In some cases, participants joined the pilot tests and provided feedback and recommendations prior to formal data collection (Berquez et al. 2011; Bernard and Norbury 2023; Biggs and Hacker 2021; Gallagher et al. 2019; Kishida et al. 2022; Munoz‐Baell et al. 2008; Studts et al. 2022).

Whilst some studies relied on a single form of PPI activity such as consultations, others utilised combinations of different approaches. For instance, the combined approach was used by some authors, including both focus groups to review the participant‐facing materials and surveys by posts to collect broad representative insights (e.g., Cooke and Millard 2018).

Despite some studies not providing detailed meeting locations, a proportion of studies specified the places where the activities took place; these activities were either conducted in‐person (e.g., Nielson et al. 2020), online (e.g., Wischmann et al. 2024) or a combination of both due to the COVID pandemic influences (e.g., Vickers et al. 2021). To gather broad opinions from different stakeholders with various perspectives, PPI contributors’ views and opinions were also collected via surveys and online forums (e.g. Kishida et al. 2022).

Topics of PPI activities also varied across studies. Six studies included the contributors’ responses in data reports, which were mostly informative studies about interventions for students with complex communication needs (Biggs and Hacker 2021), outcomes from therapy for school‐aged children who stutter (Cooke and Millard 2018; Gallagher et al. 2019), information to support children who stutter in educational settings and facilitators and barriers in screening procedures for developmental language difficulties (Berquez et al. 2011; Julien et al. 2021; Singer et al. 2020). PPI contributors also evaluated a web‐based intervention to support the social‐emotional well‐being of children who are deaf and hard of hearing (Kishida et al. 2022), a virtual reality game for children and adolescents with bilateral cochlear implants (Vickers et al. 2021), and a prototype of an informative app for the neurological impacts of paediatric hearing loss (Wischmann et al. 2024).

#### 3.5.2 Roles of PPI Contributors Mapped Onto the Stage of Involvement

Not all studies have involved PPI at all stages of research. As indicated in the guidance provided to researchers aiming to implement PPI in their research by NIHR (2024), there are seven stages where PPI contributors can be involved in the research cycle. Table 5 shows the stage of involvement identified from the included studies.

**TABLE 5:** The stage of involvement of each included study.

	Identifying and Prioritising	Commissioning		Designing & managing	Undertaking	Disseminating research	Implementing research	Evaluating impact
Munoz‐Baell et al. (2008)				✓	✓			
Berquez et al. (2011)	✓			✓	✓			✓
Cooke and Millard (2018)	✓			✓	✓			
Francis et al. (2018)				✓	✓	✓	✓	✓
Hall et al. (2019)	✓							
Buckeridge et al. (2019)				✓				
Gallagher et al. (2019)	✓			✓				
Sweeney et al. (2020)	✓			✓				
Nielson et al. (2020)						✓		✓
Singer et al. (2020)	✓			✓	✓	✓		
Biggs and Hacker (2021)	✓				✓			
Julien et al. (2021)	✓			✓				
Wilkinson et al. (2021)	✓			✓	✓	✓		✓
Vickers et al. (2021)				✓	✓			✓
Singer et al. (2022)	✓			✓	✓			
Kishida et al. (2022)	✓			✓		✓		✓
Studts et al. (2022)				✓	✓		✓	✓
Bernard and Norbury (2023)				✓				
Christopulos and Redmond (2023)	✓			✓				
Alsebayel et al. (2024)	✓			✓	✓			**✓**
Wischmann et al. (2024)				✓				

Most papers involved PPI members at the initial stage of research. For instance, they helped with reviewing the study materials to ensure their appropriateness (Cooke and Millard 2018). Moving to the next stage of research, according to NIHR (2024), commissioning requires researchers to incorporate perspectives of public members and patients into the review process and funding applications, such as reviewing the research proposal and acting as co‐applicants in the grant applications. However, it is worth noting that none of the studies reported the commission role of PPI contributors. This absence may reflect the fact that the included studies were initiated by academic researchers based on pre‐defined research agendas, with limited opportunities for PPI contributors to influence priority‐setting or funding decisions. What is more, stakeholders’ contributions as documented in publications became less frequent during the later stages of the research cycle, particularly in relation to dissemination, implementations, and evaluations. Namely, only five studies reported incorporating PPI contributors’ input into dissemination activities (Francis et al. 2018; Nielson et al. 2020; Singer et al. 2020; Studts et al. 2022; Wilkinson et al. 2021), and just two studies described implementing research findings in collaboration with PPI members (Francis et al. 2018; Studts et al. 2022), eight studies outlined evaluations on PPI (Berquez et al. 2011; Francis et al. 2018; Nielson et al. 2020; Wilkinson et al. 2021;Vickers et al. 2021; Kishida et al. 2022; Studts et al. 2022; Alsebayel et al. 2024). The evaluation of the impact of PPI that was reported in these eight papers was undertaken by the researchers.

The stage of involvement may depend on the frameworks and methodologies of PPI (Greenhalgh et al. 2019). Some authors adopted the Delphi approach to collect a broad range of opinions to identify the research or intervention priorities and then rated and ranked the relative importance of pre‐generated views. In other cases, PPI members took on more intensive roles such as co‐developing interventions or co‐authoring study materials.

### 3.6 Cycle of Tokenisms

#### 3.6.1 Lack of Support to PPI Contributors

Only two studies provided financial or material compensation to PPI members (e.g., gift vouchers), reflecting on the effort and recognition of contributors’ time. Specifically, in one study, two PPI contributors received a thank‐you letter and a gift voucher (Buckeridge et al. 2019). In Nielson et al.’s (2020) study, contributors were provided with a meal before the start of a 90‐min panel meeting. However, the remaining studies did not mention or document any compensation towards PPI contributors or their participants, which could indicate either an omission in reporting or that no compensation was provided to PPI contributors. This notable gap in transparency might be a barrier impeding patient or public involvement in the research procedure.

Given the communication barriers the targeted population may encounter, two studies reported on communication aids towards accommodating PPI contributors’ communication needs using the draw‐and‐tell technique (Gallagher et al. 2019) or hired sign language interpreters (Munoz‐Baell et al. 2008). Since many studies engaged focus groups and collaborative consultations as the format of their public involvement, it is important to moderate and facilitate the power dynamics during the discussion. However, only four studies reported that their focus group was facilitated or moderated by one of the authors (Christopulos and Redmond 2023; Gallagher et al. 2019; Nielson et al. 2020; Vickers et al. 2021).

#### 3.6.2 Level of Involvement

According to the NIHR (2024) Guidance on Public Involvement for Researchers, there are four broad approaches towards involving members of the public and patients in research: consultation, collaboration, co‐production, and user‐controlled involvement. These range from one‐directional input to bi‐directional shared decision‐making procedures, with increasing levels of partnership and power‐sharing between researchers and public contributors. The level of involvement of each study is summarised in Table 6.

**TABLE 6:** Levels of involvement of included papers in chronological order.

	Level of involvement
Munoz‐Baell et al. (2008)	Collaboration
Berquez et al. (2011)	Collaboration
Cooke and Millard (2018)	Consultation
Francis et al. (2018)	Collaboration/Co‐production
Hall et al. (2019)	Consultation
Buckeridge et al. (2019)	Consultation
Gallagher et al. (2019)	Consultation
Sweeney et al. (2020)	Consultation
Nielson et al. (2020)	Consultation
Singer et al. (2020)	Collaboration
Biggs and Hacker (2021)	Consultation
Julien et al. (2021)	Collaboration
Wilkinson et al. (2021)	Collaboration
Vickers et al. (2021)	Consultation
Singer et al. (2022)	Consultation
Kishida et al. (2022)	Consultation
Studts et al. (2022)	Consultation
Bernard and Norbury (2023)	Consultation
Christopulos and Redmond (2023)	Consultation
Alsebayel et al. (2024)	Co‐production
Wischmann et al. (2024)	Consultation

In most studies (*n* = 14), PPI was limited to consultation—the lowest level of involvement. In these cases, researchers sought the views of PPI contributors to inform aspects of the research; however, final decision‐making authority remained solely with the research team.

Only four studies adopted a collaborative approach, where researchers formed ongoing partnerships with PPI contributors and shared decision‐making responsibilities. Two studies reported engaging in co‐production, which involved joint efforts between researchers and service users across all stages of the research cycle, including the co‐creation of knowledge. Notably, no studies met the criteria for user‐controlled research—the deepest level of involvement, where public and patient contributors actively direct and manage the research process.

The evidence suggests that most studies predominantly operated at a shallow level of involvement. For example, one study explicitly stated that researchers retained final authority over the design of interventions (Julien et al. 2021). Furthermore, it remains unclear to what extent PPI contributors’ input was actually used to shape the research. While many studies claimed that individuals with lived experience helped refine participant‐facing materials, the specific changes made were not reported.

#### 3.6.3 Reporting of PPI Activities

To assess the reporting standard of PPI component in each study, GRIPP‐2 short‐form (GRIPP‐2‐SF; Staniszewska et al. 2017) was used. This is an internationally standardised reporting guideline for PPI activities in health and social care research. The checklist is given in Table 7. Generally, most studies reported information required in the GRIPP‐2 checklist, including aims of PPI (*n* = 16), methods of PPI (*n* = 18), impact on study results (*n* = 15), comments on PPI's impacts on the study (*n* = 13), and reflections on PPI experience (*n* = 12). Although the PPI was reported in each study, the level of details was inconsistent across different studies, for instance some studies just used a few sentences to describe the PPI component.

**TABLE 7:** GRIPP2‐SF checklist of each included study.

	Aim	Methods	Study results	Discussion and conclusions	Reflections/critical perspective
Munoz‐Baell et al. (2008)	✓	✓			
Berquez et al. (2011)	✓	✓	✓	✓	✓
Cooke and Millard (2018)	✓	✓	✓	✓	✓
Francis et al. (2018)		✓		✓	
Hall et al. (2019)	✓	✓	✓		✓
Buckeridge et al. (2019)		✓			
Gallagher et al. (2019)	✓	✓	✓	✓	
Sweeney et al. (2020)	✓		✓		
Nielson et al. (2020)	✓	✓	✓	✓	
Singer et al. (2020)	✓	✓	✓	✓	✓
Biggs and Hacker (2021)	✓	✓	✓	✓	✓
Julien et al. 2021)	✓	✓	✓	✓	✓
Wilkinson et al. (2021)		✓		✓	✓
Vickers et al. (2021)	✓	✓	✓		✓
Singer et al. (2022)	✓	✓	✓	✓	✓
Kishida et al. (2022)	✓	✓	✓		
Studts et al. (2022)	✓	✓			
Bernard and Norbury (2023)		✓			
Christopulos and Redmond (2023)	✓	✓	✓	✓	✓
Alsebayel et al. (2024)	✓	✓	✓	✓	
Wischmann et al. (2024)	✓	✓	✓	✓	✓

The language used to refer to PPI varied across the included studies. Besides ‘*public and patient involvement*’ (Buckeridge et al. 2019; Francis et al. 2018; Hall et al. 2019), PPI practice was implied from methodological approaches which were Participatory Action Research framework (Munoz‐Baell et al. 2008), Delphi study (Berquez et al. 2011; Cooke and Millard 2018; Singer et al. 2020), an Appreciative Inquiry‐Based Approach (Gallagher et al. 2019), the Design Council's Double Diamond approach (Design Council, 2007; Singer et al. 2022), and collaborative participatory and human centred research design (Wischmann et al. 2024).

Other studies used a descriptive phrase to refer to the PPI, including community engagement (Biggs and Hacker 2021; Nielson et al. 2020; Studts et al. 2022), collaborative work (Christopulos and Redmond 2023; Julien et al. 2021), stakeholder or user involvement (Kishida et al. 2022; Vickers et al. 2021; Wilkinson et al. 2021), and participatory design (Alsebayel et al. 2024). In some studies, PPI activities were conducted in the form of a pilot study to revise data collection materials (Studts et al. 2022).

### 3.7 Evaluations on the Impact of PPI

Many studies credited PPI input with refining research tools and procedures, as well as enhancing the clinical relevance of the work. The impact of involving patients and members of the public in research can generally be categorized into two themes: the impact on the research process itself and the clinical impact. Notably, several studies did not explicitly report any outcomes resulting from PPI involvement. This lack of detailed reporting represents a gap in the literature, limiting our understanding of how PPI contributes to research and practice.

#### 3.7.1 Research Impact

The impact of PPI on research was widely documented. A commonly‐reported benefit across studies was the enhancement of research material appropriateness. Pilot studies and focus groups were frequently used to review participant‐facing documents, making the materials more relevant and accessible to the target population. Another recurring theme was improved participant engagement and study feasibility. Some authors noted that involving patients or public members facilitated recruitment since PPI contributors were often connected with supportive communities. In a few instances, PPI input led to new insights that altered the direction of the research, for example, by incorporating outcome measures suggested by parents (Vickers et al. 2021).

In addition to increasing relevance, PPI was also reported to foster more inclusive and collaborative group dynamics, ensuring equitable opportunities for contribution (Vickers et al. 2021). Despite the involvement of diverse stakeholders, some authors acknowledged that it would have been better practice to involve children and young people with SLH difficulties directly in the research process (Alsebayel et al. 2024; Biggs and Hacker 2021; Christopulos and Redmond 2023; Singer et al. 2022; Wilkinson et al. 2021).

#### 3.7.2 Clinical Impact

Clinical practicalities of PPI of the included studies span four key domains: patients’ experience, stakeholder engagement, treatment and intervention development, and diagnostic innovation. By directly informing the materials to be sensitive to patients’ preferences and needs, resources that were informed by PPI involvement were likely to positively impact the overall well‐being of children and adolescents with SLH difficulties (Berquez et al. 2011; Nielson et al. 2020).

Also, PPI facilitated more collaborative and transparent communication between healthcare providers and stakeholders, including parents and educators. Many studies reported that involving parents in the research process improved the final discussions about treatment goals and decisions for the patients (Biggs and Hacker 2021; Cooke and Millard 2018; Francis et al. 2018; Hall et al. 2019; Sweeney et al. 2020). Moreover, PPI supported the co‐development of training materials for healthcare professionals, especially those working closely with children and adolescents with SLH difficulties, thereby promoting more inclusive and family‐centred care (Gallagher et al. 2019; Singer et al. 2022; Wilkinson et al. 2021). In addition, the active involvement of children and their stakeholders enabled the identification and prioritisation of desired therapy outcomes (Biggs and Hacker 2021; Cooke and Millard 2018) and ensured that the interventions were both evidence‐based and user‐informed (Julien et al. 2021; Vickers et al. 2021). PPI was also used to refine outcome measures, which therefore enhanced their relevance and responsiveness in evaluating the effectiveness of treatment (Cooke and Millard 2018; Sweeney et al. 2020).

Finally, PPI was important in the development and refinement of screening criteria and tools for SLH difficulties in children and adolescents. Screening tools developed through PPI were designed to be more accessible, child‐friendly, and reflective of a diverse population (Alsebayel et al. 2024). In other studies, PPI helped to identify practical barriers to screening implementation, allowing for the design of early detection strategies (Christopulos and Redmond 2023; Alsebayel et al. 2024).

## 4 Discussion

### 4.1 Summary of the Current Findings

Given the steadily growing interest in PPI over the past few decades, there is a need to examine how it is implemented in niche fields such as childhood communication difficulties, where meaningful involvement may also be shaped by the complex role of key communication partners (e.g., parents, teachers, or speech language therapists or pathologists). This systematic review aimed to assess both the quality and impact of PPI in research related to childhood communication difficulties. A secondary objective was to use the findings to inform recommendations for good PPI practice in future research. To date, there has been no consistent conceptualization of PPI, with its interpretation varying across countries and research contexts, largely due to cultural differences. To guide our review, we adopted the definition and framework provided by NIHR (2021a), a global leader in PPI, to develop a culturally grounded and methodologically rigorous search strategy.

Data were extracted from 21 eligible studies and synthesized into narratives (Popay et al. 2006). Four key synthesis domains emerged: three descriptive domains —characteristics of PPI contributors, real‐life PPI activities, and evaluation of PPI impact, and the fourth analytical domain reflecting the recurring pattern of tokenistic involvement.

Consistent with findings from previous reviews (Brett et al. 2012; Hassett et al. 2024), the majority of included studies were conducted in the UK (*n* = 9), and most were published after 2020 (*n* = 14), which may reflect the growing policy and structural support for PPI in the UK (NIHR 2021a). Furthermore, most of the included studies were rated as high quality using the Mixed Methods Appraisal Tool (MMAT; Hong et al. 2018). This may suggest that studies which included individuals with lived or living experience for the topics the research addressed tended to meet higher standards of research quality. This outcome aligns with the motivations underpinning PPI as outlined by NIHR (2024), namely, that involvement enhances the relevance, quality, and ethical integrity of research.

### 4.2 Variable Understanding, Implementation and Reporting of PPI

Generally, in line with previous literature (Dawes et al. 2024; Hassett et al. 2024; Hoddinott et al. 2018), our systematic review found that the implementation of PPI was highly variable and context‐dependent. This variability may, to some extent, have resulted from the inconsistencies in understanding and reporting of PPI components. Regarding the description of PPI, our findings showed that this heterogeneity in reporting may be shaped by contextual factors, including the country of origin, different conceptual understandings of PPI, specific research aims, and the methodological frameworks adopted. Namely, some studies employed established methodological frameworks or approaches such as participatory research, whilst others used more general descriptors such as user involvement.

Notably, in several of the studies included, PPI terminology was used interchangeably and often without clear definitions. Such inconsistencies hinder the comparability of PPI practices and their impacts across studies and impede the development of a coherent understanding of what constitutes effective PPI. Moreover, when PPI is poorly defined or vaguely reported, it risks being perceived as a tokenistic or superficial ‘tick‐box’ exercise, rather than a form of meaningful and ethical engagement. This is not to argue for a rigid and uniform model of PPI applying across all studies. As other scholars have noted, a ‘one‐size‐fits‐all’ approach fails to capture the richness and diversity of stakeholder perspectives (Greenhalgh et al. 2019). To conquer this challenge, Masterson et al. (2022) advocated for the clear articulation and operationalisation of PPI as a values‐based practice. In this regard, reporting guidelines such as GRIPP‐2 can offer valuable support for enhancing transparency, consistency, and meaningful reflection in PPI reporting.

### 4.3 Diversity and Representativeness of PPI Contributors

In our systematic review, PPI contributors included children and adolescents with communication difficulties, key communication partners such as parents, teachers, and speech‐language therapists who have direct caregiving and/or management roles, as well as public representatives, including community or organizational members. PPI participants were recruited through diverse channels, including healthcare services, support networks, public communities, prior connections with research teams, and online platforms.

Although all included studies focused on pediatric populations, only eight incorporated the direct input of children. Most studies relied on proxy perspectives provided by parents or caregivers, or other key communication partners. However, research indicates that the desire to be involved throughout the pediatric research endeavor should not be limited to parents; children themselves also express their motivations to participate (Ennis & Wykes 2013; Roulstone et al. 2016). A report by the UK Nuffield Council on Bioethics (2015) advocated for researchers to emphasize the importance of engaging children and their parents from the earliest stages of research development to demonstrating trustworthiness, transparency, and courage in the design and conduct of clinical research. In the domain of childhood communication difficulties, our review identified one study that explicitly outlined barriers to involving children, highlighting challenges such as limited literacy and understanding research content and the potential emotional burden (Singer et al. 2020). Indeed, ethical considerations concerning involving children and adolescents present additional challenges for conducting PPI studies. The existing ethical guidelines provided by NIHR (n.d.) address these ethical concerns, recommending strategies such as obtaining consent from both children and parents, allowing carers or parents to accompany children during PPI activities, and making necessary adaptations to meet children's accessibility needs.

Despite the variety of stakeholder perspectives and recruitment pathways, our systematic review validated the well‐established challenges of involving under‐represented children and young people (e.g., Greenhalgh et al. 2019), where the demographics from the under‐served population were rarely reported in our included studies. For instance, children from culturally diverse communities often grow up speaking multiple languages, and the multilingual background led to speaking English as an additional language (EAL). EAL has been historically deemed as a risk factor for speech difficulties (Byrd et al. 2016; Howell et al. 2009). As children with EAL, they may be at risk of both under‐identification and over‐identification of speech, language and communication needs, where genuine speech, language and communication needs may be missed while typical multilingual language development may be misinterpreted as disorder when assessment relies on monolingual norms or single‐language standardised measures (Boerma and Blom 2017; Ebert 2025; Lindsay and Strand 2016). These challenges may reduce access to appropriate support and limit representation in PPI activities. Future PPI work should therefore actively engage multilingual families and communities, use culturally and linguistically appropriate approaches, and avoid assuming that EAL itself indicates communication disorder.

### 4.4 Challenges of Tokenism

Tokenism remains a challenge in PPI, with critics questioning its substantive impact on shaping research quality and real‐world implementation (Biddle et al. 2021). Synthesis domains generated from the current review confirmed the tokenistic nature of many PPI practices, extending this concern into the field of childhood communication difficulties. First, we found that the depth of individual‐level involvement was generally limited. More than half of the included studies (66.7%; *n* = 14) confined PPI to the consultation stage or to early phases of the research cycle, such as identifying priorities and designing the study. Additionally, studies that involved a larger number of PPI contributors often relied on passive forms of involvement, such as data collection, rather than fostering in‐depth collaboration or co‐production. This reflects the ongoing challenge for researchers in balancing the depth of involvement with the breadth of stakeholder input.

However, larger‐scale consultation should not necessarily be viewed as less valuable than smaller‐scale collaborative involvement. The appropriate number of PPI contributors depends on the purpose of involvement. Larger groups may be useful when researchers aim to capture diverse perspectives across services, communities or geographical contexts, whereas smaller groups may be more suitable for iterative co‐design, interpretation of findings or shared decision‐making. Future studies should therefore justify the size and format of PPI according to the intended purpose, distinguish broad consultation from collaboration or co‐production, and clearly report how contributors’ input influenced research decisions.

Our findings also revealed several underlying reasons that may contribute to tokenistic involvement. First, the level of involvement was highly contextualised and largely determined by the research aims and the methodologies adopted. For instance, while some studies were conducted with the purpose of identifying research priorities within the target user population (e.g., Cooke and Millard 2018), others focussed on evaluating the interventions (e.g., Nielson et al. 2020). According to different research aims, studies may adopt various established methodologies such as the Delphi method, which is a structured, iterative method for reaching expert consensus through multiple rounds of anonymous surveys, and the James Lind Alliance (JLA) which facilitates priority‐setting partnerships (PSP) to identify and rank unanswered research questions important to patients, carers, and clinicians (NIHR, 2021; see Nygaard et al. 2019). The nature of these methodologies, which focus on setting research agendas, can lead to deeper levels of involvement (e.g., implementation) being more challenging. However, adopting the established methodologies does not mean that partnership and power‐sharing are unattainable in such contexts. As argued by Greenhalgh et al. (2019), researchers can still use these frameworks adapting to their research contexts to facilitate meaningful and collaborative involvement.

Second, the in‐depth involvement of lay contributors may be constrained due to the limited resources supporting their involvement throughout the research cycle. As our review highlighted, only two studies reported monetary incentives despite NIHR's guidance (2024) stressing its importance. Another two studies provided support to enhance children's communication accessibility. Given the already significant communication barriers faced by children with SLH difficulties, the lack of such support may further marginalize them in their endeavors to get involved meaningfully.

To address this ‘tokenistic crisis’, a clear role definition is crucial. As noted by Jayes et al. (2021), ambiguity concerning the role of public contributors can leave individuals uncertain about how best to contribute, diminishing the effectiveness of their involvement. Moreover, evaluation is a critical component of meaningful PPI, helping to ensure research is accountable and reflects appropriate use of public funds. Yet, most studies in our review described PPI outcomes from the researchers’ perspectives rather than incorporating feedback from PPI contributors themselves. Furthermore, we observed a lack of evidence regarding measurable impacts of PPI, such as improvements in participant recruitment or retention (Crocker et al. 2018), limiting our ability to assess its true effectiveness.

### 4.5 Recommendations

Challenges PPI face in real‐life practices were confirmed and extended given the particular interest in this field. To address potential challenges and facilitate future PPI implementations, recommendations corresponding to the main challenges are summarized in Table 8. As well as these recommendations, one thing to consider is the continuous release of material about PPI from NIHR. This may lead to difficulty in ensuring all advice is being followed. One thing might be to adopt versions with periodic text revisions as DSM does.

**TABLE 8:** Recommendations for better PPI practices in the field of childhood communication difficulties.

Challenges	Recommendations
Shallow involvement and tokenistic practices	Careful ethics considerations and planning following the guidance by NIHR (n.d.), especially involving children, who are deemed a vulnerable group.
	Financial and educational resources are needed to allow researchers, families, and children to engage in collaborative research (Molloy et al. 2019). For instance, The Communication Trust (2015) has published guidance on how children, young people, and their parents can be involved in decision‐making to ensure the child's views are at the centre of this. Strategies can be designed from the adaptations of existing methodologies and refer to other scholars’ implementations (see examples by PEPtalk 2, Bate et al. 2016).
	Partnering with community organisations, offering involvement opportunities in multiple languages, and compensating contributors sufficiently for their time and effort.
	Provide PPI contributors with sufficient background information due to the complex nature of the research topics. Accommodate communication needs for contributors who are patients with lived experiences, particularly in childhood SLH difficulties.
	Match the number and format of PPI contributors to the purpose of involvement. Larger groups may be appropriate for broad consultation or priority‐setting across diverse contexts, while smaller groups may support active collaboration, co‐design and shared decision‐making. Researchers should clearly report the rationale for their chosen approach and the extent to which contributors influenced decisions.
Conflicts in stakeholders’ perspectives	Weight input from different stakeholders according to relevance to the research question and insist on scientific rigor and research needs (i.e., what is worth investigating). In reports, be clear about the conflicting stakeholders’ perspectives and the rationale of the decision‐making process.
Unclear about the outcomes of PPI	Important to evaluate the impact and outcomes of PPI, not only from researchers’ perspective, but also from PPI members.
	Use existing frameworks to evaluate the outcomes and impact of PPI, such as PiiAF (Popay et al. 2014).
Confusion and inconsistency in reporting	Adhere to PPI reporting standards used in research, such as GRIPP‐2 (Staniszewska et al. 2017). Utilise available resources and frameworks. (e.g., researchers need to be aware of specific activities in which PPI contributors can take part (NIHR 2021a) and possible roles of PPI contributors in different research stages using the Involvement Matrix (Smits et al. 2020). Demonstrate the incorporation of PPI throughout the research protocol where relevant in addition to responses in PPI‐ related sections.

### 4.6 Limitations and Future Research Directions

This review has several limitations. First, the systematic review was guided by the NIHR's definition and framework for PPI. This is comprehensive and contextually relevant within the UK but may not fully capture culturally specific practices or interpretations of PPI in other international contexts. As a result, some of the review's interpretations and recommendations may lack cultural sensitivity or global applicability. Second, the literature search was conducted based on titles and abstracts only, which introduces the possibility of omitting relevant studies where PPI was discussed solely in the main text and not explicitly mentioned in the title or abstract. Third, grey literature, such as organisational reports, policy documents, and unpublished studies, was not included. This may have excluded valuable work, particularly since PPI activities are often reported in non‐peer‐reviewed formats. Furthermore, PPI contributors were not involved in the design or conduct of this review, meaning that our research process did not benefit from the very input it sought to evaluate (Pollock et al. 2021).

To address these limitations and fill the gaps identified in the current review, future research should adopt more comprehensive search strategies, including full‐text screening and grey literature inclusion, to provide a more holistic understanding of PPI practices. In 2024, the NIHR (2024b) published its first *Strategic Commitments for Public Partnerships*, outlining a national vision for improving collaboration with patients, service users, carers, and the public between 2025 and 2030. To echo this call, there is an urgent need for the field to enhance the quality of PPI practices and embed them more meaningfully across the research cycle. To fill current evidence gaps, future studies should investigate the barriers and facilitators that influence PPI contributors’ ability to engage in research, particularly from the perspectives of underserved or marginalized groups (Snape et al. 2014). Additionally, further exploration of PPI initiatives documented in the grey literature would provide a richer and more diverse evidence base, better reflecting the breadth of current PPI activity in the field of childhood communication difficulties.

## 5 Conclusions

In conclusion, the systematic review showed that PPI in childhood speech, language, and hearing difficulties has advanced significantly, with the growing interest and number of publications in the recent 5 years. The synthesized evidence shows a range of involvement examples, from children who stutter identifying the intervention priorities in speech language therapies to deaf PPI contributors shaping technology research. These efforts have led to more relevant, person‐centred research and empowered those involved. At the same time, our review highlighted challenges such as confusion and heterogeneity in PPI concepts, representativeness issues, tokenistic practices, lack of support given to lay contributors, and limited impact evaluation. All of these emphasize that PPI in the future needs continuous attention to lead to improvement. Accordingly, we provided recommendations for future research in the realm of childhood communication difficulties before, during, and after the study. Ongoing research is needed to fill evidence gaps and refine methods for involving all voices (including the children's involvement and underserved populations such as children with EAL) to harness PPI as a means to improve not only research outcomes but ultimately the lives of children and young people with communication difficulties and their families.

## Funding

This review received no specific grant from any funding agency in the public, commercial or not‐for‐profit sectors.

## Ethics Statement

Ethics statement: Institutional ethics approval (6252/002) was awarded by UCL graduate school before the study began.

## Conflicts of Interest

All authors state that they have no conflict of interest concerning this manuscript.

## Supporting information




**Supporting Table S1**: Study characteristics of included papers in chronological order.

**Supporting Table S2**: Extracted PPI data from included papers in chronological order.

## Data Availability

The current article is a systematic review that involves the synthesis of data from previously published studies. The data extracted from included studies are provided in the supplementary materials. The search strategies, data extraction domains and PRISMA checklist are also available as supplementary files.

## Appendix A

### A.1

PRISMA checklist

**Table:**

Section and Topic	Item #	Checklist item	Location where item is reported
**TITLE**			
Title	1	Identify the report as a systematic review.	Title
**ABSTRACT**			
Abstract	2	See the PRISMA 2020 for Abstracts checklist.	Abstract
**INTRODUCTION**			
Rationale	3	Describe the rationale for the review in the context of existing knowledge.	Introduction—The current study
Objectives	4	Provide an explicit statement of the objective(s) or question(s) the review addresses.	Introduction—The current study
**METHODS**			
Eligibility criteria	5	Specify the inclusion and exclusion criteria for the review and how studies were grouped for the syntheses.	Methods—Eligibility criteria
Information sources	6	Specify all databases, registers, websites, organisations, reference lists and other sources searched or consulted to identify studies. Specify the date when each source was last searched or consulted.	Methods—Search strategy
Search strategy	7	Present the full search strategies for all databases, registers and websites, including any filters and limits used.	Methods—Search strategy
Selection process	8	Specify the methods used to decide whether a study met the inclusion criteria of the review, including how many reviewers screened each record and each report retrieved, whether they worked independently, and if applicable, details of automation tools used in the process.	Methods—Study selection
Data collection process	9	Specify the methods used to collect data from reports, including how many reviewers collected data from each report, whether they worked independently, any processes for obtaining or confirming data from study investigators, and if applicable, details of automation tools used in the process.	Methods—Data extraction
Data items	10a	List and define all outcomes for which data were sought. Specify whether all results that were compatible with each outcome domain in each study were sought, and if not, the methods used to decide which results to collect.	Methods – Table 2; Table S1
Data items	10b	List and define all other variables for which data were sought, such as participant and intervention characteristics and funding sources. Describe any assumptions made about any missing or unclear information.	Methods – Table 2; Tables S1 and S2
Study risk of bias assessment	11	Specify the methods used to assess risk of bias in the included studies, including details of the tool used, how many reviewers assessed each study and whether they worked independently, and if applicable, details of automation tools used in the process.	Methods—Methodological quality assessment
Effect measures	12	Specify for each outcome the effect measure used in the synthesis or presentation of results.	N/A
Synthesis methods	13a	Describe the processes used to decide which studies were eligible for each synthesis.	Methods—Data extraction; Methods—Eligibility criteria
Synthesis methods	13b	Describe any methods required to prepare the data for presentation or synthesis, such as handling of missing summary statistics or data conversions.	Methods—Data extraction; Methods—Synthesis methods
Synthesis methods	13c	Describe any methods used to tabulate or visually display results of individual studies and syntheses.	Methods—Synthesis methods; Table 3, 4, 5, 6; Tables S1 and S2
Synthesis methods	13d	Describe any methods used to synthesize results and provide a rationale for the choices. If meta‐analysis was performed, describe the models, methods to identify the presence and extent of statistical heterogeneity, and software packages used.	Methods—Synthesis methods
Synthesis methods	13e	Describe any methods used to explore possible causes of heterogeneity among study results, such as subgroup analysis or meta‐regression.	N/A
Synthesis methods	13f	Describe any sensitivity analyses conducted to assess robustness of the synthesized results.	N/A
Reporting bias assessment	14	Describe any methods used to assess risk of bias due to missing results in a synthesis arising from reporting biases.	N/A
Certainty assessment	15	Describe any methods used to assess certainty or confidence in the body of evidence for an outcome.	N/A
**RESULTS**			
Study selection	16a	Describe the results of the search and selection process, from the number of records identified in the search to the number of studies included in the review, ideally using a flow diagram.	Results—Study characteristics; Figure 1
Study selection	16b	Cite studies that might appear to meet the inclusion criteria, but which were excluded, and explain why they were excluded.	Figure 1
Study characteristics	17	Cite each included study and present its characteristics.	Results—Study characteristics
Risk of bias in studies	18	Present assessments of risk of bias for each included study.	Results—Methodological quality assessments
Results of individual studies	19	For all outcomes, present, for each study, summary statistics for each group where appropriate and an effect estimate and its precision, ideally using structured tables or plots.	N/A
Results of syntheses	20a	For each synthesis, briefly summarise the characteristics and risk of bias among contributing studies.	Results—Study characteristics; Methodological quality assessment; Tables S1 and S2
Results of syntheses	20b	Present results of all statistical syntheses conducted. If meta‐analysis was done, present the summary estimate and its precision and measures of statistical heterogeneity. If comparing groups, describe the direction of the effect.	N/A
Results of syntheses	20c	Present results of all investigations of possible causes of heterogeneity among study results.	N/A
Results of syntheses	20d	Present results of all sensitivity analyses conducted to assess the robustness of the synthesized results.	N/A
Reporting biases	21	Present assessments of risk of bias due to missing results arising from reporting biases for each synthesis assessed.	N/A
Certainty of evidence	22	Present assessments of certainty or confidence in the body of evidence for each outcome assessed.	N/A
**DISCUSSION**			
Discussion	23a	Provide a general interpretation of the results in the context of other evidence.	Discussion—Summary of the current findings
Discussion	23b	Discuss any limitations of the evidence included in the review.	Discussions
Discussion	23c	Discuss any limitations of the review processes used.	Discussions—Limitations and future research directions
Discussion	23d	Discuss implications of the results for practice, policy, and future research.	Discussions—Recommendations; Discussions—Limitations and future research directions
**OTHER INFORMATION**			
Registration and protocol	24a	Provide registration information for the review, including register name and registration number, or state that the review was not registered.	Methods—Protocol registration
Registration and protocol	24b	Indicate where the review protocol can be accessed, or state that a protocol was not prepared.	Methods—Protocol registration
Registration and protocol	24c	Describe and explain any amendments to information provided at registration or in the protocol.	N/A
Support	25	Describe sources of financial or non‐financial support for the review, and the role of the funders or sponsors in the review.	Funding
Competing interests	26	Declare any competing interests of review authors.	Competing interests
Availability of data, code and other materials	27	Report which materials are publicly available and where they can be found, including template data collection forms, data extracted from included studies, data used for analyses, analytic code, and any other materials used in the review.	Data availability
