# Supplementary material for: A Systematic Review on Patient and Public Involvement in Research on Childhood Communication Difficulties
Source: Int J Lang Commun Disord. 2026 Jun 27;61(4):e70281. doi: 10.1111/1460-6984.70281 (PMC13309805; doi:10.1111/1460-6984.70281)
Supplement: Supplementary file 1 — Supporting Table S1: Study characteristics of included papers in chronological order. [file JLCD-61-0-s001.docx]

**Table S.1**

*Study characteristics of included papers in chronological order*

| \| **Study** \| **Country** \| **Study design** \| **Research questions** \| **Participant characteristics** \| **Type of difficulties** \| **Confirmation of diagnosis of difficulties** \| **Comorbidities** \| **Quality of assessment** \| \| --- \| --- \| --- \| --- \| --- \| --- \| --- \| --- \| --- \| \| Munoz-Baell et al. (2008) \| Spain \| Quantitative \| Examines how the usually separate strands of school health promotion and d/Deaf education might be woven together and illustrates research with Deaf community members that involves them and gives their perspective. \| 210 participants from organisations for d/Deaf community \| Deaf \| N/A \| Participants are from various organisations for d/Deaf community \| Medium \| \| Berquez et al. (2011) \| United Kingdom \| Mixed: Delphi study \| Explores what information children, parents and education staff feel would be important to know to support a child who stutters in the educational environment, in order to develop appropriate resources. \| Children who stutter (CWS) aged 7–11 (n = 25)  young people who stutter aged 12–18 (n = 27)  parents of children and young people who stutter aged 2–18 (n = 67)  members of the education workforce (n = 35). \| Stuttering \| PWS were recruited from the MPC specialized for stuttering. \| N/A \| High \| \| Cooke & Millard (2018) \| United Kingdom \| Mixed: Delphi study \| Examines what school- aged children who stutter consider to be the most important outcomes from therapy. \| 25 children who stutter (7-14 years) who had been referred to the Michael Palin Center for Stammering (MPC) who were able to read and write in English and the language level was aimed to be understood by 6-year-olds and above. \| Stuttering \| PWS were recruited from the MPC specialized for stuttering. \| N/A \| High \| \| Francis et al. (2018) \| United Kingdom \| Quantitative RCT \| Determines the clinical and cost-effectiveness of a 7-day course of oral steroids in improving hearing at 5 weeks in children with persistent OME symptoms and current bilateral OME and hearing loss demonstrated by audiometry. \| Children aged 2–8 years, with symptoms of hearing loss attributable to OME for at least 3 months \| Hearing loss associated with otitis media with effusion \| a diagnosis of bilateral OME made on the day of recruitment and audiometry-confirmed hearing loss \| N/A \| High \| \| Hall et al. (2018) \| United Kingdom \| Mixed \| Scopes current service provision across England for management of otitis media with effusion (OMS) and hearing loss in children with Down syndrome.  Explores professional decision‐making about managing otitis media with effusion and hearing loss.  Examines patient and public views on the direction of future research. \| 21 audiology services in England took part in the evaluation; 10 professionals participated in the qualitative study \| Hearing loss associated with otitis media with effusion \| The hearing loss was identified by the audiology services in England \| Down syndrome (DS) \| Low \| \| Buckeridge et al. (2019) \| United Kingdom \| Qualitative \| Explores adolescents’ everyday experiences of communication following acquired brain injury. \| 6 verbal adolescents (11-18 years) who were at least one year post a diagnosed ABI \| Communication difficulties \| Paediatric acquired brain injury (ABI) \| N/A \| High \| \| Gallagher et al. (2019) \| Ireland \| Qualitative \| Develops a conceptual model to guide collaborative practice engaging key stakeholders in the co-design of their ideal speech and language therapy service and support in school. \| 29 participants in total: SLTs (N = 8), teachers (N = 5), parents (N = 9) and children with DLD (N = 7). \| DLD \| N/A \| N/A \| High \| \| Sweeney et al. (2020) \| United Kingdom, Ireland \| Quantitative RCT \| Evaluates the speech, activity and, participation outcomes of Parent Led, Therapist Supervised, Articulation Therapy (PLAT) compared with routine speech therapy intervention in parent–child dyads. \| 44 children, aged 2.9–7.5 years  Parents, in the parent-trained group (n = 23) \| Speech difficulties associated with cleft palate \| N/A \| N/A \| Medium \| \| Nielson et al. (2020) \| USA \| Qualitative \| Determines whether a community engagement approach can provide feedback for implementation of valuable measures to improve the outcome of a clinical trial \| parents of children born with congenital cytomegalovirus-induced and subsequent sensorineural hearing loss. The panel of experts was 6 males and 7 females. \| Hearing loss \| the Collaboration & Engagement Team from Primary Children's hospital recruited parents of children born with CMV and subsequent sensorineural hearing loss. \| N/A \| High \| \| Singer et al. (2020) \| Netherlands \| Mixed: Delphi study \| Develops a definition and operationalization of communicative participation in 2- to 8-year-old children with language difficulties. \| 10 parents, 5 young adults with language difficulties, 7 teachers and assistants, 11 speech-language pathologists, 4 clinical linguists, 4 children psychologists, and 6 clinical researchers. \| Language difficulties \| Clinical researchers were recruited based on their research interests and experience.   Other participants were recruited via letters on social media, including professional, parental, and patient networks. \| Autism spectrum disorder; Intellectual disability \| High \| \| Biggs & Hacker (2021) \| USA \| Qualitative \| Understands intervention priorities for students with complex communication needs. \| 4 parents of children with complex communication needs in kindergarten through 12th grade, or who was 18–21years of age and received school-based transition services; 6 special education teachers; 4 paraprofessionals; 5 school-based SLPs. \| Complex communication needs \| child who used any form of aided or unaided AAC as a primary communication mode (e.g., gestures or body movements, eye gaze, facial expressions, manual signs, picture symbols, speech-generating device [SGD]) \| Autism spectrum disorder; Deaf-blindness; Developmental delay; Intellectual disability; Multiple disabilities; Orthopedic impairment; Other health impairment; Traumatic brain injury; Visual impairment. \| High \| \| Julien et al. (2021) \| Canada \| Qualitative \| Constructs a logic model to design a new intervention program  Improves the communication and social skills of adolescents with DLD in a secondary school setting. \| Adolescents with developmental language difficulties (n = 2) and their parents (n = 2), professionals in practice settings (n = 9), and members of the research team (n = 6) \| DLD \| N/A \| N/A \| Medium \| \| Wilkinson et al. (2021) \| United Kingdom \| Mixed \| Develops a training package to support healthcare staff improving the communication with children with communication difficulties and their families.  Examines the feasibility of implementation in the ward. \| 5 senior clinicians for recruiting participants from the study.  9 facilitators (three Practice Educators, two Nurses, one Deputy Sister, one trainer for Newly Qualified Nurses, one Learning Disability (LD) Nurse and one Child Disability Clinical Specialist) delivered sessions.  In total, 123 healthcare staff participated. \| Disabled children with communication difficulties \| Children were identified from the ward. \| N/A \| High \| \| Vickers et al. (2021) \| United Kingdom \| Qualitative \| Develops a package of virtual-reality games (BEARS, Both EARS) to train spatial hearing in young people (8–16 years) with bilateral cochlear implants using an action-research protocol \| spatial hearing in young people (8–16 years) with bilateral cochlear implants \| Hearing loss \| N/A \| N/A \| High \| \| Singer et al. (2022) \| Netherlands \| Mixed \| Describes a co-design process that led to the development of a physical artefact that can support SLTs to engage parents of children with DLD in collaborative goal-setting. \| Dutch SLT–practitioners, SLT– researchers, co-design researchers, co-design students (n=4) and parents of children with DLD (n=70 out of total 145) \| DLD \| N/A \| N/A \| High \| \| Kishida et al. (2022) \| Australia \| Quantitative non-randomized \| Describes outcomes of a pilot study of the web-based resource intervention to support deaf and hard of hearing students’ social–emotional well-being \| Children who are Deaf and Hard of Hearing (DHH), their parents, Teachers of the Deaf, and other community stakeholders \| Hearing loss \| All students enrolled in mainstream schools in Grades 4–6 (aged 10–12 years) with mild to profound sensorineural hearing loss (with a severity greater than 20 dB), together with their parents and classroom teachers \| 4 children with anxiety disorder. \| High \| \| Studts et al. (2022) \| USA \| Mixed \| Describes a program of stakeholder-engaged research adapting and assessing behavioral parent training with caregivers of young Deaf and Hard of Hearing children followed in hearing health care, aimed at reducing inequities in access to behavioral parent training \| caregivers of Deaf and Hard of Hearing children \| Hearing loss \| local and regional hearing health care clinics \| N/A \| Medium \| \| Bernard & Norbury (2023) \| UK \| Quantitative \| Investigate on the factors associated with symptoms of anxiety and depression in children who stutter \| 35 children and adolescents who stutter aged between 8-13 years (M = 128 months, SD = 20.4 months) and their parents participated in the study. \| Stuttering \| Young people lived with stuttering were recruited from the Youth Panel in a charity supporting children and young people who stutter in the UK. \| N/A \| High \| \| Christopulos & Redmond (2023) \| USA \| Mixed \| Identifies and understands contextual factors that facilitate or hinder the implementation of universal screening for DLD. \| 157 participants from the school district (69 SLPs + 88 general education teachers). \| DLD \| N/A \| N/A \| High \| \| Alsebayel et al. (2024) \| USA \| Mixed \| Developed a child-friendly app that supported the assessment of speech difficulties while engaging children in gamified experiences. \| Preschool children, SLPs \| N/A \| N/A \| N/A \| High \| \| Wischmann et al. (2024) \| Denmark \| Qualitative \| Examines how to use emerging technologies to enhance the understanding of the neurological impact of pediatric hearing loss.   Develops an app and evaluates its ease of use and the understanding of neurology by all types of stakeholders and end-users. \| 8 parents with children (aged 2-12 represented all degrees of hearing loss and hearing technologies), 13 internal stakeholders and 14 external stakeholders \| Hearing loss \| the ENT & Audiology department of Copenhagen Hearing and Balance Centre (CHBC), largest paediatric ENT & Audiology department in Denmark. \| N/A \| Medium \| |  |  |  |  |  |  |  |  |  |
| --- | --- | --- | --- | --- | --- | --- | --- | --- | --- | --- | --- | --- | --- | --- | --- | --- | --- | --- | --- | --- | --- | --- | --- | --- | --- | --- | --- | --- | --- | --- | --- | --- | --- | --- | --- | --- | --- | --- | --- | --- | --- | --- | --- | --- | --- | --- | --- | --- | --- | --- | --- | --- | --- | --- | --- | --- | --- | --- | --- | --- | --- | --- | --- | --- | --- | --- | --- | --- | --- | --- | --- | --- | --- | --- | --- | --- | --- | --- | --- | --- | --- | --- | --- | --- | --- | --- | --- | --- | --- | --- | --- | --- | --- | --- | --- | --- | --- | --- | --- | --- | --- | --- | --- | --- | --- | --- | --- | --- | --- | --- | --- | --- | --- | --- | --- | --- | --- | --- | --- | --- | --- | --- | --- | --- | --- | --- | --- | --- | --- | --- | --- | --- | --- | --- | --- | --- | --- | --- | --- | --- | --- | --- | --- | --- | --- | --- | --- | --- | --- | --- | --- | --- | --- | --- | --- | --- | --- | --- | --- | --- | --- | --- | --- | --- | --- | --- | --- | --- | --- | --- | --- | --- | --- | --- | --- | --- | --- | --- | --- | --- | --- | --- | --- | --- | --- | --- | --- | --- | --- | --- | --- | --- | --- | --- | --- | --- | --- | --- | --- | --- | --- | --- | --- | --- | --- | --- | --- |
