# Supplementary material for: A Systematic Review on Patient and Public Involvement in Research on Childhood Communication Difficulties
Source: Int J Lang Commun Disord. 2026 Jun 27;61(4):e70281. doi: 10.1111/1460-6984.70281 (PMC13309805; doi:10.1111/1460-6984.70281)
Supplement: Supplementary file 2 — Supporting Table S2: Extracted PPI data from included papers in chronological order. [file JLCD-61-0-s002.docx]

**Table S.2**

*Extracted PPI data from included papers in chronological order*

| **Study** | **Term of PPI** | **Characteristics** | **Number of contributors** | **Compensation** | **Recruitment** | **PPI activities** | **Impacts on research** | **Evaluation of PPI** | **Clinical Implications** |
| --- | --- | --- | --- | --- | --- | --- | --- | --- | --- |
| Munoz-Baell et al. (2008) | Community participation; Participatory Action Research framework | Deaf community stakeholders | N/A | N/A | Contacted PPI contributors' national umbrella organisation (CNSE), consultation with national organ- isations involved in d/Deaf education, literature searches and the Internet | The questionnaire was initially piloted with a sample of key informants representing each targeted group. Particular emphasis was placed on maintaining a simple and accessible style to ensure that the questions were easily comprehensible to the intended audience, especially members of the Deaf community.  PPI contributors met with the research teams regularly with the aid of sign language interpreters to provide direction to the research process from a Deaf community perspective, and to incorporate feedback and lessons learned back into the research design, the process and the data analysis.   Working definitions of health, health promotion, health- promoting schools, Deaf community issues, Deaf bilingual education and participatory research were discussed and agreed upon early in the research process and revisited at several stages of the project. | suggest effective indicators in the planning for health-promoting schools initiatives for Deaf children. | "enhanced the ability to reconcile different agendas, build consensus and decision-making, engage in meaningful dialogue and develop a common strategy to attain goals. Partnership, participation and trust-building are viewed by the extended research team as the keystone of the project and as its main strength." | N/A |
| Berquez et al. (2011) | Delphi study | Children who stutter aged 7–11 (n = 25)  Young people who stutter aged 12–18 (n = 27)  Parents of children and young people (n = 67)  Members of the education workforce (n = 35) | 154 | N/A | Families attended the Michael Palin Centre for Stammering Children (MPC)   Public members accessing the British Stammering Association (BSA) via www.stammering.org  Teachers and support staff from schools in and around London | Stage 2 Formulation of the question: Developed and piloted the questions before distributing to a wider panel of experts. Each focus group generated a variety of potential questions and, through collaborative discussion, reached consensus on the question they believed would elicit a diverse range of responses.  Stage 3 Statement generation: 94 participants generated statements in response to the questions devised by each of the focus group.   Stage 5 Rating of the statements: The resulting statements were distributed to participating experts within the relevant groups, who were asked to rate each statement on a 5-point Likert scale based on the perceived importance of its inclusion. | PPI contributors and stakeholders reflected on different perspectives and identified key messages to be communicated to the education workforce about stuttering. | Increase the content validity of resulting materials.  Include more breadth of opinion which could be communicated across the panels by including PPI contributors with different living and professional experiences.  Potential sample biases for recruiting experts from a single venue. | The resulting resources have the potential to impact positively on the well-being of a highly vulnerable group of children and teenagers across England. |
| Cooke & Millard (2018) | Delphi study | Expert panel: children who stutter aged 8 to 14 (n=25) who had been referred to the Michael Palin Center for Stammering (MPC).  Focus group: researchers and clinicians who specialized in stuttering (n=5). | 30 | N/A | Expert panel: convenience sampling by contacting school-aged CWS from the MPC.  Focus group: N/A | Focus group: designed the meaningful question and prompted questions for CWS  Stage 2 Formulation of the question: The question for the current study was developed in consultation with a focus group comprising five researchers and clinicians who are specialists in stuttering. Two children gave feedback on the phrasing of the question. Consequently, prompt questions were developed to encourage the participant to think across several domains, including home, school, and friendships.  Stage 3 Statement Generation: 18 children who stutter generated the responses.  Stage 5 Rating of the statements: 15 children who stutter rated the importance of each statement on a 5-point scale. | PPI contributors identified the important treatment outcomes from the therapy. | Enabled CWS’s views to be included in consideration and generating relevant ideas.  Improved the study quality (i.e., high face validity).  Reduced the influences of others’ opinions or group dynamics. | 21 most important therapy outcomes identified by CWS.  Results can be further developed into a tool to identify therapy's priorities & evaluate outcomes from the child's perspective.  Emphasized the need to involve parents & teachers in therapy. |
| Francis et al. (2018) | Patient and Public Involvement | A mother who has a child with long-standing OME (n = 1)  Other PPI representatives who had personal experience of children with OME | 1 in Trial Steering Committee  Others in Independent Data Monitoring Committee | N/A | N/A | Reviewed parent and child information sheets, provided feedback on the trial protocol and guidance on recruitment strategies.  Involved in the analysis and dissemination of study results.  Sarah Jones (Patient and Public Representative) contributed to the study design, study implementation in terms of PPI and final approval of the report. | Successful recruitment.  Hearing tests are relatively objective, related to the resolution of the underlying pathology and to functional status. | N/A | Informed discussions between parents and clinicians and decisions about parents' preferred treatment. |
| Hall et al. (2018) | Patient and Public Involvement | Family members (n=21)  Adults with Down syndrome (n=10)  Charity representatives (n=2) | 33 | N/A | N/A | Three PPI groups were held and attended by 1) family members consisting of twelve mothers and one grandmother where eight parents provided feedback outside the groups; 2) ten adults with DS; and 2 charity representatives.   Identified research topics and priorities relating to OME and hearing loss that are important to service users.  Presented the topics arising from the research.  Discussed future research priorities. | The directions of future research were identified by PPI contributors from different perspectives. | N/A | Suggested future directions to improve treatment decisions for children with Down syndrome and OME: + Develop shared decision-making tools for parents + Establish core outcomes for research + Decide on key clinical and quality measures for services + Develop evidence to guide future management guidelines for children with DS. |
| Buckeridge et al. (2019) | Patient and Public Involvement | Adolescents with acquired brain injury (n=3) | 3 | A letter of thanks and gift voucher | N/A | Planned the research.  Two adolescents with ABI were consulted about the design and giving feedback used to develop the participant-facing materials and question guide for the interview. They also commented on the invitation letter and practicalities of the interview, such as seating and refreshments.  Supported the development of an adolescent-friendly version of research findings. | Making the study materials more relevant | N/A | N/A |
| Gallagher et al. (2019) | Appreciative inquiry-based approach | SLTs (n = 8)  Teachers (n = 5)  Parents (n = 9)  Children with DLD (n = 7) | 21 | N/A | Parents and children were recruited via a national support network for parents with DLD using snowballing techniques. Practitioners were recruited through professional bodies and established clinical networks via email/phone contact | A pilot session (1 SLT, 1 teacher, and 1 parent) to refine the topic guide for the focus groups.  Parents and practitioners in focus groups discussed the ideal speech and language therapy service and supports to schools. All focus groups were facilitated by the first author. An observer was present at each session to document any non-verbal interactions and/or actions that occurred between participants and the facilitator, using a standard observation checklist.  Conducted semi-structured interviews with children with DLD for similar topics; used draw-and-tell technique. The children’s comprehension of tasks was assisted by employing augmentative methods of communication. | Policy: to reinforce the status of the child as a ‘being’ in their own right and provide clear guidance around issues of ‘voice' (that children should be able to give influential input)   Research: the need to consider different methodologies such as sociological approaches to the study of the classroom. the need to continue to increase awareness about DLD and to lobby for the necessary resources for SLTs to be able to work in a meaningful way in schools. | A small number of stakeholders, not representative of the views of teachers, SLTs, parents or children with DLD in general  Provided a rich description of the ideal service and supports, carefully chosen because of participants' particular knowledge and experience in relation to SLT services and supports | Practice: practitioners need to learn the skills necessary to ‘listen’ to children with DLD. Practitioners need to be given the opportunity to learn about these techniques, understand their rationale and to use them as part of their everyday interactions with children. |
| Sweeney et al. (2020) | N/A | Parents | N/A | N/A | N/A | Parent focus group (see Supplementary Document A) | Many parents were anxious and frustrated with the lack of access to speech and language therapy services and wanted to be empowered to help their children improve their speech.  The assessment protocol was largely successful, with minor adaptations recommended (Sweeney et al., 2017). | N/A | parents highlighted the need for adequate support during a home-based programme  intervention should be evaluated in a randomized controlled trial, with adaptations to the age range, inclusion/exclusion criteria and parental support (Sweeney et al., 2017) |
| Nielson et al. (2020) | Community engagement | Seven mothers and six fathers of children born with CMV and subsequent sensorineural hearing loss (n = 13). | 13 | A meal was provided for panel participants prior to the start of a studio meeting. | The Collaboration & Engagement Team from Primary Children's hospital recruited parents of children born with CMV and subsequent sensorineural hearing loss. | A brief presentation was given by the primary investigator about the virus and the present trial followed by a discussion led by a neutral moderator.   Community experts discussed reasons for enrolling their child in the clinical trial, suggested ways to make participation more appealing, offered communication strategies for the research team, and provided feedback on the social media ad and website. | Identified parents' need to clearly understand the risks and benefits of participating in the trial.  In response to the parents' comments, two educational videos were created: one on the virus and one on the clinical trial. | From the studio panel, parents gave valuable input regarding the ValEar trial to enhance patient experience and participation.   Since the CE Studio was completed at the beginning of the trial, it is difficult to compare the impact of the meeting on patient recruitment.   Parents preferred to have quicker access to and more engagement with the primary investigator. The research team did not take any specific steps to address this feedback, although methods allowing physician access is worth investigating. | Communication throughout the trial is important factor to improve the patient experience, whether directly with the provider or indirectly through social media. |
| Singer et al. (2020) | Delphi study | Parents with children with language difficulties (n = 10)  Teachers and teaching assistants (n = 7)  Young adults with language difficulties (n = 5) | 22 | N/A | Letters via social media | Cyclical process, in which panel members gave input and opinions repeatedly, and moderators (i.e., the first two authors) summarized opinions within the group.  For each subsequent round, controlled anonymized feedback was provided to the Delphi panel by presenting summaries of the data from the previous round and a new survey that was developed based on collected data.  Four rounds: 1) open-ended brainstorm questions to identify topics; 2) members rated concept definitions using 7-point Likert scale; 3) members answered clarifying questions and substantiate their answers; 4) members rated redrafted definitions using 7-point Likert scale and provided feedback based on summarized data of Round 2 and 3.   To facilitate participation of many different stakeholders, researchers used easy-to-understand language when writing items and drafting definitions. | The study resulted in a definition and a 33-item operationalization of children’s communicative participation. | Panel members' opinion were effectively combined into group consensus.  Lack of direct interaction between members (online study).  Small panel size.   The Delphi process is anonymous, allowing panel members with different backgrounds to have an equal voice. | The operationalization gave clinicians and parents clear examples of children’s communication in daily life. The items concern a broad range of behaviors, from understanding someone’s nonverbal messages to clarifying one’s communicative needs to other people. |
| Biggs & Hacker (2021) | Engage stakeholders | Parents of children with communication needs (n = 4)  Professionals including teachers (n = 6), paraprofessionals (n = 4), and school-based SLPs (n = 5), who have worked with a student who had complex communication needs within the same school year as the start of the study. | 19 | N/A | Electronic and print flyers through social media pages and community resources | Prior to data collection, interview guides and the handout were piloted with several parents and professionals; minor wording changes were made based on their feedback.  In-depth interviews.  Participants were involved in intermediate-level member checks where asked to identify if the findings matched with their perspectives. | Intervention priorities were identified from different perspectives. | Not diverse demographically (mostly females and White)  Should included other stakeholders (general education teachers, education administrators, fathers, and individuals with complex communication needs themselves) | For children and families: This may provide a useful tool to discuss important goals with professionals.  For educators and service providers, it is important for professionals to equally attend to utilizing strategies that target students’ opportunities and supports across their natural environments. |
| Julien et al. (2021) | Co-construction; Collaborative work; Advisory committee | Adolescents with DLD (n = 2) and their parents (n = 2)  Professionals including SLPs (n = 4), teachers (n = 3), members of school boards (n = 2), occupational therapist (n = 1), 1 guidance counsellor (n = 1) | 9 | N/A | Convenience recruitment: recruited on a voluntary basis from the larger study sample through the partnerships established by the principal investigator for the main study. | In-person PPI consultations were conducted. PPI contributors were invited to the four stages, including 1) Problem identification; and 2) Definition of the need for an intervention and available resources; 3) Goal establishment; 4) Outlining of the objectives and desired outcomes. | The logic model of an intervention was designed for young adolescents with DLD in a secondary school setting. | Convenience sample may not be representative.  The subjectivity was included because the main choices were made by researchers. | The logic model was designed to inform an intervention.  Recommendations for speech-language pathology research teams wishing to use LM development were made. |
| Wilkinson et al. (2021) | Stakeholder involvement; Steering group | Parents of disabled children with communication difficulties.  Paediatricians and nurses and other ward staff.  Disabled students from a local college. | 6 parents  The number of paediatricians and disabled students were not specified. | N/A | Parents were recruited from the PenCRU Family Faculty.  Disabled students were recruited from a local college. | Parent carers identified the need; develop and evaluate the training, including featuring in the training video. They were also consulted throughout the study on research design, delivery and reporting.  Disabled students from a local college designed the poster, which acted as an aide memoire on the ward to remind staff of the key messages around communication that are taught in the training. | The involvement of parent carers profoundly influenced the content of the training to include real family experiences and deliver messages they feel are important. | Standardised way: Evaluation on staff: Questionnaires were sent pre- and post-study to healthcare staff, which evaluated the frequency, confidence of contact with patients and the support provided by the hospital.  Evaluation on hospital practices and procedures: continuation to use the materials and to train staff.  Absence of disabled children as key stakeholders  Researchers: were guided by the experiences of parents carers to identify and deliver the key messages | Co-developed by stakeholders, the training materials for healthcare staff communicating with disabled children who have communication difficulties and their families, which facilitate to gather more information about children’s needs. |
| Vickers et al. (2021) | Co-create; user involvement; Action research | Young people and young adults using bilateral cochlear implants (9-18 years old), family and friends, teachers | Phase I: 18 children & adolescents   Phase II: 16 children & adolescents  Phase III: teachers of the deaf, 6 children & adolescents | N/A | Phase I: 10 participants at a school for deaf and hard-of-hearing (DHH) children, who volunteered to help in response to an advert. The other eight (n = 3 male, n = 5 female) were recruited from an advert circulated by a charity and attended a meeting held in London.  Phase II: Older children and teenagers from mainstream and special schools (n = 6 male, n = 5 female) with an additional 5 younger participants (n = 2 male, n = 3 female)   Phase III: N/A | Involved multiple focus groups for feedback, reflection, and critical appraisal.  Phase I: Two groups of CI children joined in-person discussions to identify the difficulties of living with bilateral CIs and to understand what the acceptable interventions were.  Phase II: Children reviewed of the practicalities of the prototype to understand the practical limitations of BEARS. The feedback were provided based on the ease of use of BEARS and in an in-person event.   Phase III: The teachers and clinicians discussed in an online meeting to review the tools from a clinical and educational perspective.  The bilateral CI group reviewed the details of the software tools and components online as well. Each group was made up of two bilateral CI users who were matched to work well together (based on teacher opinion), one facilitator and one notetaker. | The notes on the difficulties were grouped together and set priorities for the project. | Professionals, young bilateral CI users in primary school and those in secondary school are separated to ensure that all participants felt comfortable to engage and contribute to the discussions.   Primary-school-aged child was accompanied by a caregiver to change the dynamics of the focus group.  Lack of diversity in participants' backgrounds. | A logic model was created by the research team to underpin the planned multi-modal BEARS virtual reality auditory training intervention.  Determine if the BEARS training suite would be appropriate for a wider age range of CI users, understand the practical limitations.  BEARS training suite was finalized for use in a clinical trial. |
| Singer et al. (2022) | Co-design | Co-design students (n=4)   Parents of children with DLD (n=59) | 63 | N/A | SLT–practitioners each invited six parents of a child with DLD (in total 48) from their caseload  4 co-design students were recruited via a tutor of an international co-design minor  Parents in the usability study were recruited via the SLT–practitioners | Stage 1 Discover: SLT–practitioners had have a short interview with at least five parents about their child’s well-being and the importance of certain values in life (e.g., health, relationships and education). Parents’ views on speech and language therapy and responsibilities in the therapy process were also incorporated.  A team of co-design students used the personas, categories and items, and design guidelines to develop a first concept.   Usability testing of the tool: Four SLTs invited parents to discuss their child’s communicative participation problems. SLT–researchers used the tools with the parents and interviewed them about their experiences. After the first test round, the comments of the SLTs and parents, as well as parts of the video recordings of the conversations were fed back to the co-design researchers who adjusted the tool | Parents' feedback was used to improve the prototype and manual. | Parents’ input on the development of tool was not sought.  Parents’ involvement was limited to an interview on their children’s wellbeing and values in life via participating SLT–practitioners, and in the usability testing. should have included parents as equal partners in the co-design process.   Based on experiences in other projects where parents are part of the research team, researchers were convinced that parents could have made a valuable contribution in any co-process aimed at improving the care for their children.  From children's perspective, children aged 2-7 are too young to participate a co-design process with written instruction and communication, but researchers think that there is an urgent need to develop tools and methods for shared goal-setting with children that incorporates their unique perspectives, aspirations, and challenges. | Interviews helped SLT-practitioners build up an understanding of, and empathize with parents' needs, emotions, motivations, and ways of thinking.  Resulted in the co-design of a physical artefact called ‘ENGAGE’, which promotes family-centered care and shared decision-making between parents & SLTs. |
| Kishida et al. (2022) | Consumer and community involvement | Students who are DHH (n = 10; Grades 6–7, aged 12– 13 years), their parents (n = 10), Teachers of the Deaf (n = 14)  Classroom teachers (n = 10) and other mainstream school staff members (n = 2)   Translation Advisory Group: 20 organizations’ representatives from DHH community, professional bodies to support DHH (e.g., speech pathology, audiology, government and nongovernment schools, community health services, and Aboriginal health services)  Pilot study: 37 students, 37 parents, 40 mainstream teachers | 66 | N/A | Students enrolled in mainstream schools in Grades 4–6 (aged 10–12 years) | Forums, workshops, and interviews for students, parents, and teachers of the DHH students to identified social-emotional needs of students who are DHH, strategies to address the needs, roles of parents, additional resources  Student ambassadors attended structured workshops to identify the social–emotional needs of students who are DHH.  A parent forum was held concurrently with the student ambassador forum.  Two separate work- shops were held for school-based professionals working to support students who are DHH.  Semi-structured interviews for classroom teachers and school staff.  Consulted the Translation Advisory Group for strategic advice to the project and to ensure translation of findings to the community.  Provided feedback on the developed website product before it was released | Identified parents' needs and developed web-based resource to support DHH students' emotional & social well-being  Highlighted the need for further and more extensive research to help families whose children are DHH  A community organization has disseminated the web resource to allow public access (https://wafdc.org.au/ your-child-their-school/). | Actively engaged parents of children who are DHH and other key stakeholders in the co-design and development of the web-based resource | N/A |
| Studts et al. (2022) | Pilot study | 12 biological parents (age 18 years or older) of DHH children (ages 2–5 years) who used hearing aids and/or cochlear implants | 12 | N/A | local and regional hearing health care clinics | Caregivers completed multiple instruments about sociodemographic background, child behaviors, parenting, language development, and parent satisfaction.  Caregivers were grouped by child’s type of hearing device and randomized in a 1:1 ratio to intervention (an abbreviated three-session family check-up) or attention–control (a family healthy eating intervention for caregivers).  Caregivers also gave open-ended feedback. | Feasibility of the study procedures was supported.  Caregivers appreciated learning new skills and receiving insights and direction from the interventionists. | N/A | Follow-up interview revealed that the lack of representation of DHH children and the lack of inclusion of common behavioral scenarios faced by their caregivers could limit the value of the intervention. |
| Bernard & Norbury (2023) | N/A | Young people who stutter aged 16–25 years who advocate for greater public awareness about stuttering, and the charity's Board of Trustees. | N/A | N/A | Young people lived with stuttering were recruited from the Youth Panel in a charity supporting children and young people who stutter in the UK. | Members from the Youth Panel participated in the piloting of the survey. | Ensure the appropriateness of the participant-facing materials. | N/A | N/A |
| Christopulos & Redmond (2023) | Collaborative research | Related Services administrators (n=2)  Kindergarten teachers (n=2) | 4 | N/A | The school district that authors have previously collaborated with to recruit PPI contributors who were directly involved in either the screening administration or its planning phases in the previous study. | Semistructured in-person interviews were conducted to identify the barriers and facilitators. Focus groups were conducted in person and moderated by the first author.   Qualitative findings and survey items were sent to PPI contributors to ensure these are relevant. |  | Acquire detailed and nuanced understanding of the topic.   The sample might not be representative.   Views from children and families with lived experiences were not incorporated. | Identified and generalized relevant barriers and facilitators in the screening for DLD, facilitating the universal implementation of it. |
| Alsebayel et al. (2024) | Participatory design; Co-create | Preschoolers with typical development (n=9)  Game designer (n=3)  Computer vision engineers (n=2)  Game developer (n=1) | 13 | N/A | Preschoolers: childcare center at a university | Focus group: 2 CVEs, 3 game designers, and 2 SLPs validated findings in the initial observational study and explored any additional challenges, needs, or requirements to support pediatric speech assessment based on video data.  Concept co-design: 1 CVE, 1 game developer, and 2 game designers helped brainstorm themes for the games.  Prototype: 1 CVE, 1 game developer, and 2 game designers participated in prototype development.  9 preschoolers played the game and gave feedback in two user studies to evaluate the game. | Developed an app that prompts children to perform speech assessment tasks in a gamified setting. | The sample was not representative of children with speech difficulties. | The first step towards developing a low-cost, noninvasive, child-friendly speech assessment tool that could clinically be used to support objective assessment of motor speech difficulties to measure facial movements.  If successful, the tool may allow early detection of speech difficulties. |
| Wischmann et al. (2024) | A collaborative participatory and human centred research design | Parents of children with hearing loss  (n =8) | 8 | N/A | ENT & Audiology department of Copenhagen Hearing and Balance Centre (CHBC), which is the largest paediatric ENT & Audiology department in Denmark. | 13 stakeholders from CHBC and 8 end-users evaluated the prototype by completing the questionnaire online. | Both stakeholders and end-users evaluated the prototype in a systematic way, whose feedback were based on to make the final adjustments to the app.  The participatory and human centred research design was highly applicable to use in paediatric audiological research.  The app will be useful in providing information for parents of children with hearing loss. | Both stakeholders and end-users gave constructive feedback. | The app will have substantial clinical significance (not specified) |
